# A consistent approach to the genotype encoding problem in a genome-wide association study of continuous phenotypes

**DOI:** 10.1371/journal.pone.0236139

**Published:** 2020-07-15

**Authors:** Sunhee Kim, Chang-Yong Lee

**Affiliations:** The Department of Industrial and Systems Engineering, Kongju National University, Cheonan, South Korea; University of Sao Paulo/Luiz de Queiroz Agriculture College, BRAZIL

## Abstract

In this study, we suggested a hypothesis test method that was robust to different genotype encodings in a genome-wide association analysis of continuous traits. When the population stratification is corrected for using a method based on principal component analysis, ordinally (or categorically) encoded genotypes are adjusted and turn into continuous values. Due to the adjustment of the encoded genotype, the association test result using conventional methods, such as the test of Pearson’s correlation coefficient, was shown to be dependent on how genotypes were encoded. To overcome this shortcoming, we proposed a non-parametric test based on Kendall’s tau. Because Kendall’s tau deals with rank, rather than value, associations between adjusted genotype and phenotype values, Kendall’s test can be more robust than Pearson’s test under different genotype encodings. We assessed the robustness of Kendall’s test and compared with that of Pearson’s test in terms of the difference in *p*-values obtained by using different genotype encodings. With simulated as well as real data set, we demonstrated that Kendall’s test was more robust than Pearson’s test under different genotype encodings. The proposed method can be applicable to the broad topics of interest in population genetics and comparative genomics, in which novel genetic variants are associated with traits. This study may also bring about a cautious approach to the genotype encoding in the numerical analysis.

## Introduction

A genome-wide association study (GWAS) includes the statistical test of associations between genetic variants, such as SNPs, and phenotypes (or traits) of interest across samples. In the past 10 years since GWAS were first introduced, about 10,000 robust associations with disease, disorder, and other genomic traits have been discovered [1]. Detecting associations between genetic variants and traits depend on many factors such as the sample size, the frequency of the genetic variants, and the linkage disequilibrium between the observed and unknown causal variants. Besides, the population stratification (or population structure) should be taken into account to avoid spurious associations caused by the genetic differences in samples from different populations. A high variability in allele frequencies across subpopulations, for example, would be falsely associated with a phenotype.

Various methods have been proposed to correct for the population structure. The genomic control approach utilized the distribution of test statistics to estimate the inflation factor, with which the test statistics were subsequently rescaled to avoid the risk of false positives [2, 3]. An allele-frequency admixture model implemented in the program STRUCTURE used genotype data to partition samples into subpopulations [4]. A more elaborate model of mStruct [5] was proposed to include the effect of allele mutations between ancestral and current alleles. In addition, the genotype-conditional association test was implemented by a method based on the genotypes and the logistic regression [6]. Parallel to the model-based approach, a linear mixed model and its subsequent works were proposed, in which the fixed and the random effects detected the population structure and the family (or cryptic) relatedness, respectively [7–10]. Besides the model-based approaches, a dimensionality reduction method using principal component analysis (PCA) was proposed by using genome-wide markers as the variables and implemented in the software package EIGENSTRAT [11, 12]. In the presence of known or cryptic genetic relatedness, an elaborate PCA-based method of robust inference for the population structure was proposed by identifying a diverse set of unrelated samples [13, 14]. In PCA-based methods, a categorically (or ordinally) encoded genotype becomes a continuous value as a result of the correction for the population structure. A PCA-based method subtracts an amount attributable to ancestry along top principal components (PCs) from numerically encoded genotypes, via computing residuals of linear regressions. Because PCs as covariates are the axes of variation in a multilinear regression, PCA would apply greater correction to markers with larger differences in allele frequency across ancestral populations.

The genotype of a bi-allelic SNP is either homozygous or heterozygous and can be represented as a pair of alleles, e.g., *AA* and *aa* for homozygous major and minor genotypes, and *Aa* for a heterozygous genotype. As the genotype of an SNP is categorical in nature, we have freedom of encoding the genotype as long as an encoding scheme can distinguish different genotypes. A common way of genotype encoding has been an ordinal encoding, which was based on the number of different alleles present. For example, genotypes *AA*, *Aa*, and *aa* have elements coded as -1, 0, and 1, respectively. Additionally, one can count the number of minor alleles in the genotype and represent them as 0, 1, and 2. Genotype encodings {0, 1, 2} and {−1, 0, 1} correspond to measuring an additive effect at a SNP. Encoded genotype {0, 1, 2} represents the number of minor allele in the genotype and {−1, 0, 1} is associated with vanishing level of dominance. Besides the two types of genotype encodings, many other encodings are theoretically possible as long as different genotypes can be distinguished [15, 16]. As an example of other encodings, the dominance effect encodes 0 for homozygous major (*AA*) and 1 for heterozygous (*Aa*) or homozygous minor (*aa*).

Ordinally (or categorically) encoded genotypes, such as {0, 1, 2} and {−1, 0, 1}, turn to continuous values after the adjustment by a PCA-based method. The encoded genotypes are adjusted by subtracting the variations due to the difference between the subpopulations. As the number of axes of variation (or principal components) included in the adjustment becomes larger, the difference between the encoded genotype and its adjusted value tends to become greater. Because adjusted genotype values are no longer categorical, usual test methods for the association between categorical genotypes and continuous phenotypes, such as the *F*-test, cannot be applied. Instead, the associattion test based on Pearson’s correlation coefficient (hereafter, Pearson’s test for short) would be a typical test method for the association between continuous genotypes (i.e., adjusted genotype values) and continuous phenotypes. The Pearson’s test statistic is a function of Pearson’s correlation coefficient and follows a *t*-distribution under the assumption that continuous genotypes and phenotypes are distributed as independent normal distributions. Pearson’s test is a parametric test method and is also known as the test for the slope of a linear regression model. Incidentally, a *t*-test for the slope of an additive genetic model is different from Pearson’s test and has been adopted, for example, by GAPIT [17] in the association test.

The amount of a genotype adjustment depends not only on the number of principal components but on how genotypes are encoded. This means that the association test results may be affected by the genotype encoding, which raises the question of finding an adequate genotype encoding scheme. Because any genotype encoding scheme is, in principle, acceptable as long as the encoding can distinguish different genotypes, the correct genotype encoding may not exist. Thus, the best policy might be to find an association test method whose test results are robust under different genotype encodings. The genotype encoding problem also occurs in genomic selection and breeding, in which a continuous phenotype was predicted in a linear regression model [15, 16]. It was demonstrated that the genetic prediction heavily depended on the genotype encoding not only for a single marker model but for an epistasis model.

Under the assumption that a PCA-based method was adopted to correct for the population structure, in this study, we addressed the robustness of the association test methods under different genotype encoding schemes. We investigated the validity of Pearson’s test under different genotype encodings and demonstrated that the test statistic was not invariant under different genotype encodings. To alleviate, if not solve completely, the non-invariance under different genotype encodings, we proposed a test method based on the Kendall rank correlation coefficient (or Kendall’s tau) [18]. The Kendall’s tau is a statistic, which measures the ordinal association between two variables (in our case, adjusted genotype and phenotype values). The proposed method is a non-parametric test, which is known to be more suitable for ordinal data than a parametric test [19]. In this sense, the proposed method may be more appropriate than a parametric method because genotypes are generally encoded as ordinal quantities, which naturally contain the concept of ranking but have no clear numerical interpretation. When the ranking of adjusted genotype values are maintained under different encoding schemes, the test statistic can be insensitive to different genotype encodings and their adjustments.

While we provided theoretical analysis of the non-invariance of Pearson’s test under different genotype encodings, we supported Kendall’s test by demonstrating that the relative order in magnitude between a pair of adjusted genotypes was likely to be maintained. By using simulated as well as real data set, we assessed the robustness of Kendall’s test and compared with that of Pearson’s test under different genotype encodings. We empirically demonstrated that Kendall’s test was more robust than Pearson’s test in terms of the difference in the *p*-values obtained from different genotype encodings. These findings illustrated that Kendall’s test was a more consistent approach to the genotype encoding problem than Pearson’s test.

## Results

In principle, a “good” association test method should produce test results that are robust to different genotype encodings. That is, the *p*-values obtained from a good test method are insensitive within statistical fluctuation to the genotype encoding. One way to quantify the robustness was the difference in the *p*-values obtained from two different encoding schemes. To this end, we defined the difference in the *p*-values for SNP *i* as

$$\begin{array}{c} \Delta p_{i}\equiv p_{i,E_{1}}-p_{i,E_{2}}, \end{array}$$ (1)

where $p_{i,E_{1}}$ and $p_{i,E_{2}}$ were the *p*-values for SNP *i* obtained by using the genotype encodings of *E*_1_ and *E*_2_, respectively. A test method would become more robust, as it generates smaller Δ*p*_*i*_ (i.e., Δ*p*_*i*_ → 0).

**Fig 1:**
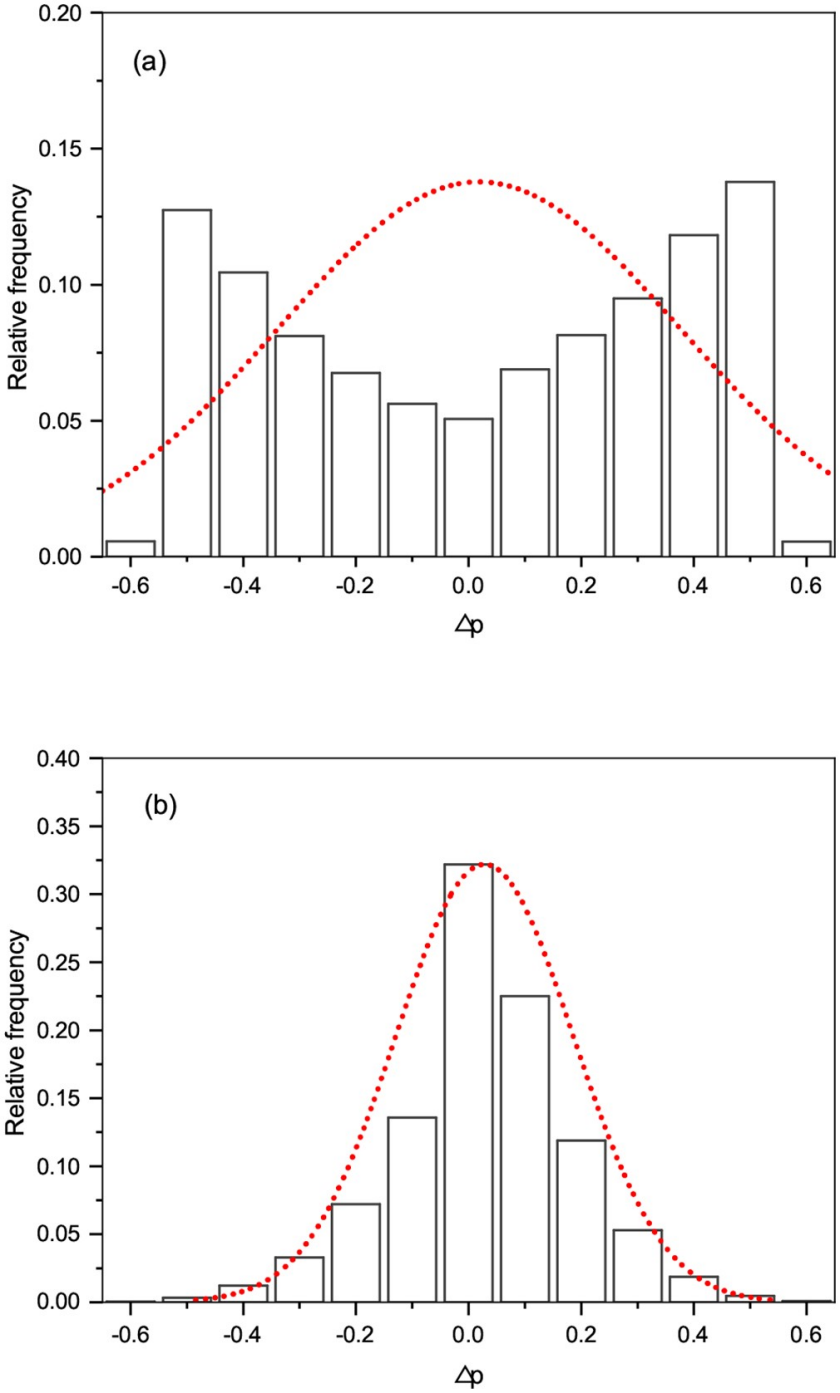
Frequency distributions of Δ*p*_*i*_ obtained by (a) Pearson’s test and (b) Kendall’s test. The dotted curves represented the corresponding normal probability distributions with zero mean and the variance estimated by measured Δ*p*_*i*_. The genotype and phenotype data were generated from the method described in Section *Generation of simulated data*. The parameters used in generating data were: the number of SNPs *m* = 200, 000, minor allele frequency *q* = 0.25, the shuffling probability *ρ* = 0.5, and the rate of variation *π* = 0.05.

Fig 1 showed a typical frequency distribution of Δ*p*_*i*_ obtained by Pearson’s and Kendall’s tests using simulated data with two types of genotype encoding, *E*_1_ = {0, 1, 2} and *E*_2_ = {−1, 0, 1}, which have been used more often than other encodings. The simulated genotypes and phenotypes data were generated by the method described in Section *Generation of simulated data*. As shown in Fig 1(a), the frequency distribution of Δ*p*_*i*_ obtained by Pearson’s test showed two peaks away from zero mean (i.e., Δ*p*_*i*_ = 0). Considering that measurement errors often had a distribution which was close to a normal distribution, Δ*p*_*i*_ obtained by Pearson’s test were highly unlikely to be statistical errors. In contrast, a typical frequency distribution of Δ*p*_*i*_ obtained by Kendall’s test had unimodal and was close to a normal distribution centered around zero mean as shown in Fig 1(b). Thus, Δ*p*_*i*_ obtained by Kendall’s test were more likely to be statistical errors than that by Pearson’s test. From this finding, we could infer that Kendall’s test could be more robust than the usual method of Pearson’s test under different genotype encodings.

### Test statistics under genotype transformation

To investigate the robustness of Pearson’s and Kendall’s tests, we examined both test statistics theoretically as well as empirically under different genotype encodings. As demonstrated in Section *Invariance and non-invariance of test statistics*, we noted that a different genotype encoding could be implemented by a combination of two types of transformations: a multiplicative and an additive transformations. We found that test statistics of both tests were invariant under the multiplicative genotype transformation, whereas Pearson’s test was not invariant under the additive genotype transformation. A detailed account of the invariance and non-invariance was given in Section *Invariance and non-invariance of test statistics*. These findings left us with the investigation of the invariance of Kendall’s test under the additive genotype transformation.

As we discussed in Section *Invariance and non-invariance of test statistics*, it was not easy to obtain an analytic expression of the changes in the Kendall’s test statistic under the additive transformation. We thus empirically investigated the test statistic under the additive transformation. To this end, we generated genotype data with genotype encoding of *E*_1_ = {0, 1, 2} and *E*_2_ = {−1, 0, 1}, which are related by an additive transformation of *E*_1_ = *E*_2_ + *β* with *β* = 1. We then evaluated $\delta_{jk}^{adj}$, the difference between a pair of samples *i* and *j* in their PCA-adjusted genotype values with *E*_2_. Similarly, we evaluated $\delta_{jk}^{\beta ,adj}$ with *E*_1_. A detailed description of method and notations including $\delta_{jk}^{adj}$ and $\delta_{jk}^{\beta ,adj}$ was given in Section *Invariance and non-invariance of test statistics*.

**Fig 2:**
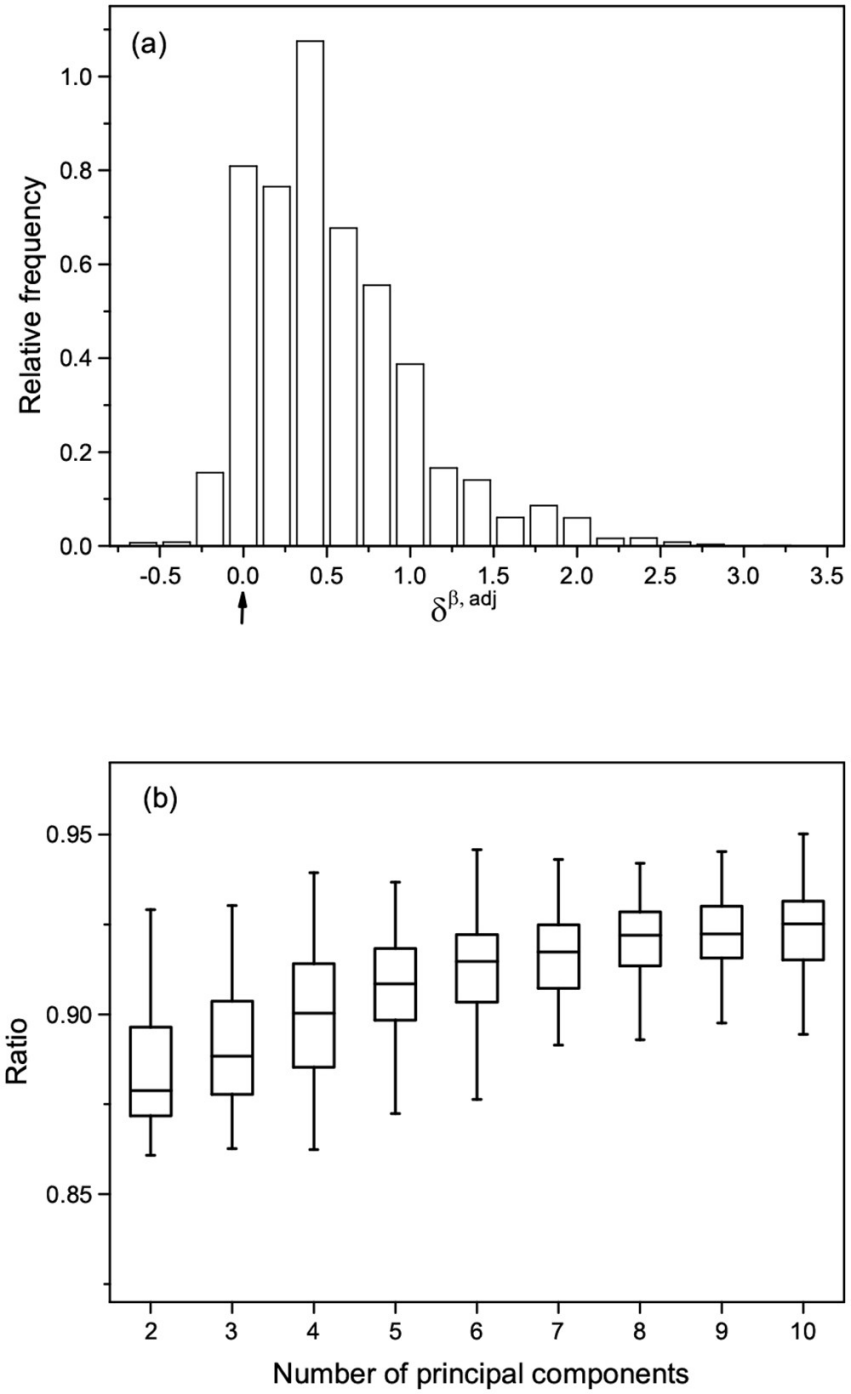
(a) The frequency distributions of *δ*^*β*, *adj*^ with the sample size 300 and the number of principal components *K* = 5, given *δ*^*adj*^ > 0. The arrow below the abscissa indicates *δ*^*β*, *adj*^ = 0. (b) Box plots of the ratio versus the number of principal components used for the genotype adjustment.

We plotted the frequency distribution of $\delta_{jk}^{\beta ,adj}$ for all pairs of samples, given $\delta_{jk}^{adj}>0$ in Fig 2(a). As we could see from Fig 2(a), the frequency distribution of $\delta_{jk}^{\beta ,adj}$ was heavily skewed toward the right around *δ*^*β*, *adj*^ = 0. This showed that when $\delta_{jk}^{adj}$ were positive most of $\delta_{jk}^{\beta ,adj}$ were also positive. Similarly, given $\delta_{jk}^{adj}<0$, the distribution of $\delta_{jk}^{\beta ,adj}$ was heavily skewed toward the left around *δ*^*β*, *adj*^ = 0 as shown in Supporting information S1 Fig. These results illustrated that Kendall’s tau was highly likely to preserve the relative order of the adjusted genotype values under the additive genotype transformation.

To quantify the degree of maintaining the relative order under the additive transformation, we estimated the ratio of the number of sample pairs that maintained their relative orders to the total number of sample pairs. We showed the ratio versus the number of principal components used for the genotype adjustment in Fig 2(b), from which we found that the ratio was in the range of [0.88, 0.93]. This demonstrated that about 88% to 93% of sample pairs maintained their relative orders under the additive genotype transformation. In addition, the ratio increased as the number of principal components used for the genotype adjustment increased. This supported that the ratio became higher as the difference between a genotype value and its adjusted value became greater. These results suggested that the ranks of adjusted genotypic values across *n* individuals were likely to be preserved after the additive transformation.

### Assessment of suggested method using simulated data

**Fig 3:**
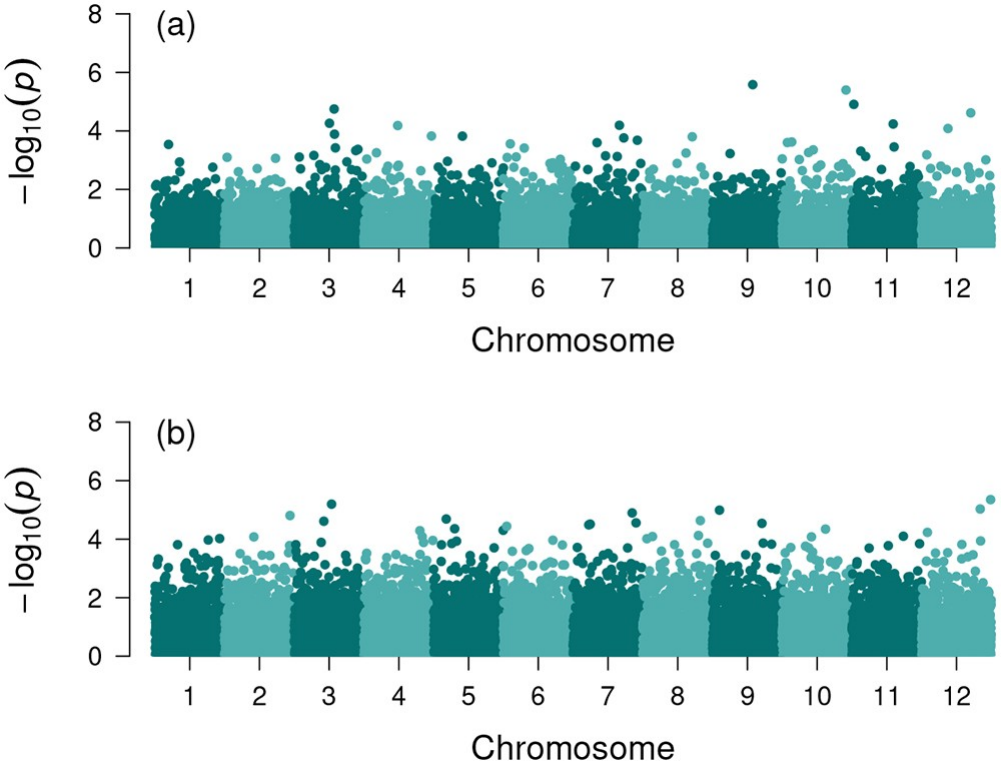
Manhattan plots of the association analysis using (a) Pearson’s test and (b) Kendall’s test. Note that we fictitiously divided simulated SNPs into 12 chromosomes for the display purpose. The used parameters are: the number of SNPs *m* = 200, 000, sample size *n* = 300, the rate of variation *π* = 0.05, and the shuffling rate *κ* = 0.4.

Having shown that Pearson’s and Kendall’s tests were invariant under the multiplicative genotype transformation, we then compared the performance of the two tests under the additive genotype transformation. To this end, we generated simulated data set as described in Section *Generation of simulated data* with two genotype encodings *E*_1_ = {0, 1, 2} and *E*_2_ = {−1, 0, 1}, which were related to the additive genotype transformation. To assess and compare the result of Kendall’s test with that of Pearson’s test, we performed a hypothesis test for each SNP *i* under the null hypothesis that adjusted genotypes $g_{ij}^{adj}$ and phenotypes *p*_*j*_ were uncorrelated (i.e., the slope of a linear regression model between adjusted genotypes and phenotypes was zero). In Fig 3, we showed typical Manhattan plots of the association analysis using both Pearson’s and Kendall’s tests with simulated data. As shown in Fig 3, although there was difference between two Manhattan plots, the difference was not easy to be characterized systematically. To analyze the difference in association results, we utilized the power of a hypothesis test, which is the probability that a hypothesis test rejects the null hypothesis when a specific alternative hypothesis is true. Higher power implies less chance of making false negative errors. Thus, the power can be a statistical measure to quantify the possibility of capturing more true marker-phenotype association.

**Fig 4:**
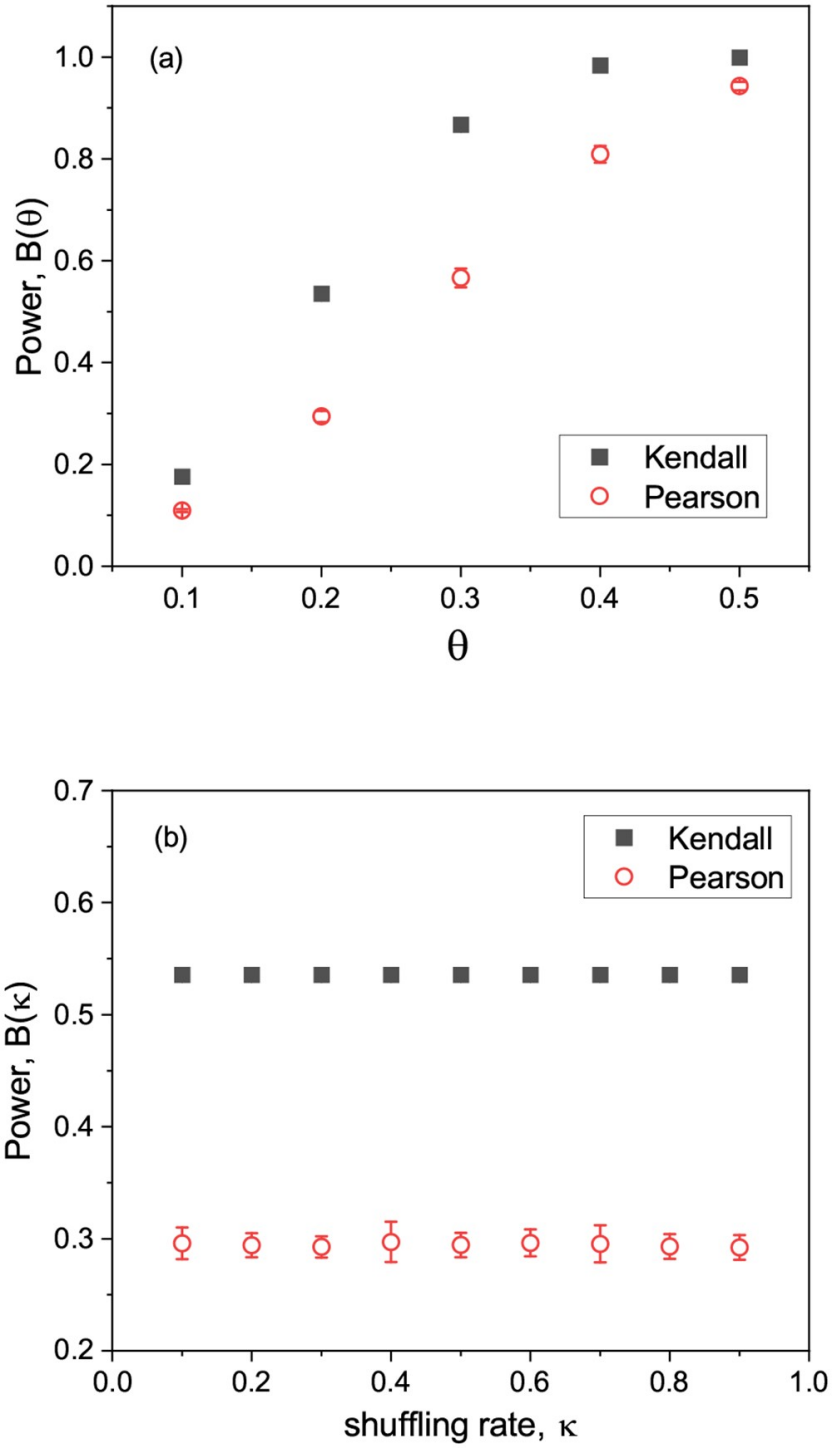
(a) The plot of estimated powers obtained from Kendall’s and Pearson’s tests with simulated data versus *θ*, specified values of the alternative hypothesis. (b) The plot of estimated powers versus the shuffling rate for a given *θ* = 0.2. Note that estimated powers from Pearson’s test were averaged over 20 SNPs and the error bars represented the standard deviations, some of which were too small to be seen. Note also that because the power from Kendall’s test depends only on the number of samples for a given *θ*, the estimated power was same for all SNPs.

Because the alternative hypothesis in our case was not an equality but the negation of the null hypothesis, we referred to the power against a specific alternative hypothesis. Fig 4 showed the power estimated by two test methods with simulated data. We found from Fig 4(a) that Kendall’s test was more powerful than Pearson’s test for all specified values of the alternative hypothesis. This result was in contrast to the general notion that a non-parametric test was less powerful than a parametric test. We obtained a similar result under different shuffling rates as shown in Fig 4(b). While the power of both tests was insensitive to the shuffling rate, Kendall’s test was more powerful regardless of the magnitude of the shuffling rate. Thus, we can conclude that Kendall’s test would generate fewer false negatives than Pearson’s test at least within the context of simulated data sets used in this study.

To investigate the robustness of both test methods, we described and quantified the frequency distribution of Δ*p*_*i*_ defined in Eq (1) in terms of two statistical measures: skewness *G*_1_ and kurtosis (or excess kurtosis) *G*_2_ [20]. Skewness is a measure of the symmetry of a frequency distribution about the mean. As skewness is closer to zero, the distribution becomes more symmetric. Skewness in our case can be used to check whether a hypothesis test method has a systematic bias in the *p*-value toward a particular genotype encoding. When skewness deviates noticeably from zero, a test method tends to produce systematically larger (or smaller) *p*-values for a particular genotype encodings than those for other encodings. Kurtosis is a measure of the concentration of probability mass in a distribution around the mean. A large kurtosis tends to have high concentration of data around the mean. In our case, as kurtosis of the distribution of Δ*p*_*i*_ around zero mean becomes larger, the corresponding test method would be more likely to generate Δ*p*_*i*_ closer to zero. This means that a test method is more robust under different genotype encodings as the corresponding kurtosis becomes larger. A detailed description of skewness and kurtosis together with their interpretation was given in Section *Skewness and kurtosis*.

**Fig 5:**
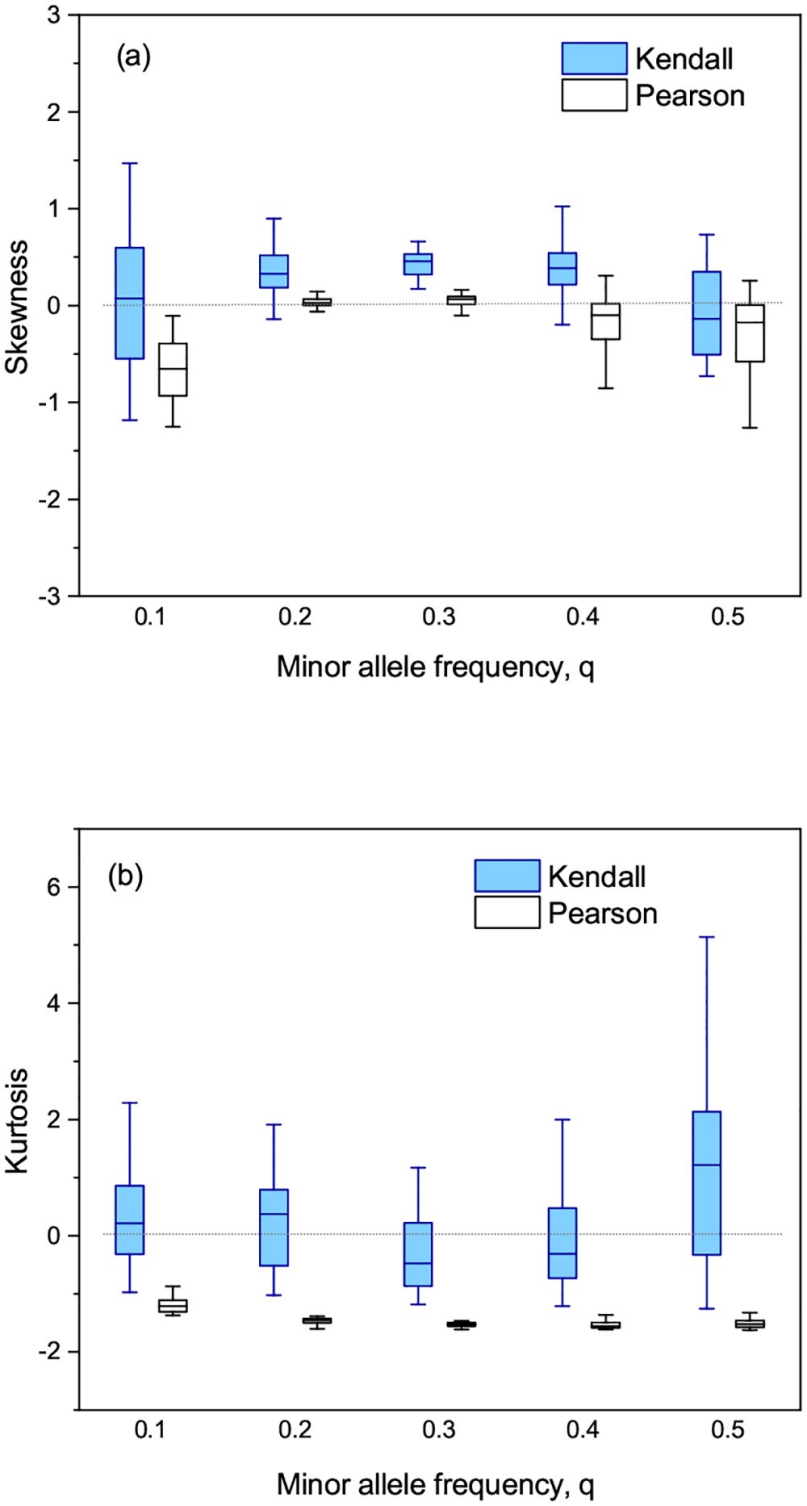
Box plots of (a) skewness and (b) kurtosis estimated from the distribution of Δ*p*_*i*_ obtained by Kendall’s and Pearson’s tests versus the minor allele frequency. The used parameters are: the number of SNPs *m* = 200, 000, sample size *n* = 300, the rate of variation *π* = 0.05, and the shuffling rate *κ* = 0.5.

We estimated skewness and kurtosis of the frequency distribution of Δ*p*_*i*_ estimated for all SNPs around zero sample mean. Fig 5 showed box plots of estimated skewness and kurtosis versus the minor allele frequency *q*. As shown in Fig 5(a), estimated skewness from Kendall’s and Pearson’s tests was fluctuating more or less around zero. This implied that the distribution of Δ*p*_*i*_ was symmetric at least moderately if not completely and that both test methods were not biased toward any particular genotype encoding. As we can see from Fig 5(b), estimated kurtosis from Kendall’s test was significantly higher than that from Pearson’s test for all minor allele frequencies. The distribution of Δ*p*_*i*_ obtained by Kendall’s test was mesokurtic (i.e., *G*_2_ ≈ 0), similar to a normal distribution as shown, for example, in Fig 1(b). In contrast, the distribution obtained by Pearson’s test was platykurtic (i.e., *G*_2_ < 0) meaning that Δ*p*_*i*_ were less concentrated around zero mean than a normal distribution as shown, for instance, in Fig 1(a). This implied that Δ*p*_*i*_ obtained by Kendall’s test was more concentrated around zero mean than that obtained by Pearson’s test. This result supported stronger robustness of the proposed test compared to Pearson’s test under different genotype encodings.

**Fig 6:**
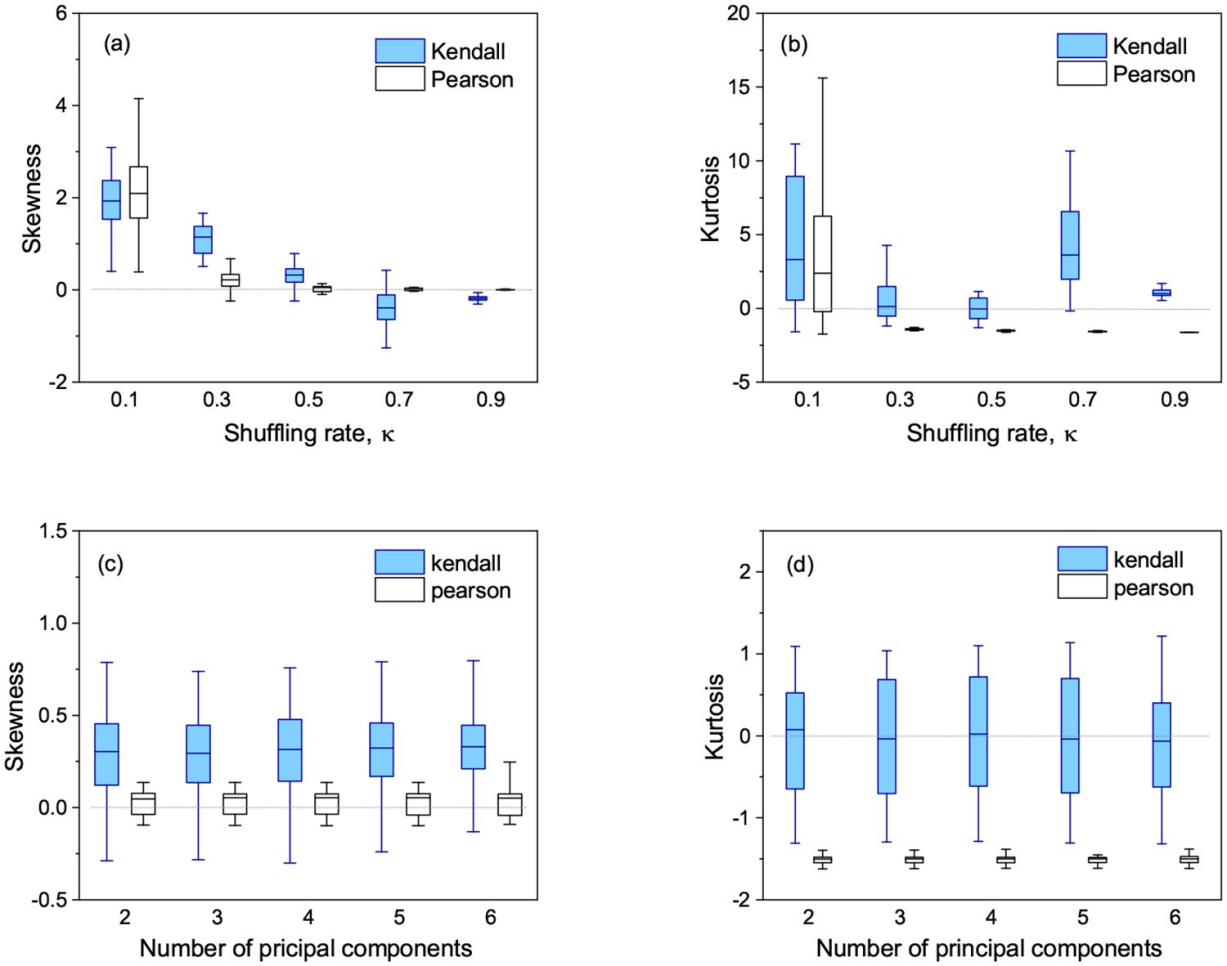
(a-b) Box plots of (a) skewness and (b) kurtosis estimated from the distribution of Δ*p*_*i*_ obtained by Kendall’s and Pearson’s tests versus different shuffling rates. The used parameters are: the number of SNPs 200,000, sample size 300, *π* = 0.05, and the minor allele frequency 0.25. (c-d) Box plots of (c) skewness and (d) kurtosis estimated from the distribution of Δ*p*_*i*_ obtained by Kendall’s and Pearson’s tests versus the number of principal components used for the genotype adjustment. The used parameters are: the number of SNPs *m* = 200, 000, sample size *n* = 300, the rate of variation *π* = 0.05, the minor allele frequency *q* = 0.25, and the shuffling rate *κ* = 0.5.

We next estimated skewness and kurtosis of the distribution of Δ*p*_*i*_ by varying the shuffling rate *κ* described in Section *Generation of simulated data*, while holding other parameters fixed. We varied the shuffling rate *κ* in generating simulated data to control the number of significantly associated SNPs with the phenotype. As shown in Fig 6(a) and 6(b), we found similar results to the above case of varying the minor allele frequency. While estimated skewness and kurtosis varied from the shuffling rate, kurtosis from Kendall’s test was higher than that from Pearson’s test for all shuffling rates. This meant that Kendall’s test generated Δ*p*_*i*_ more likely to be close to zero than Pearson’s test did. In addition, the distributions of Δ*p*_*i*_ obtained by the two tests were more or less symmetric around zero mean as estimated skewness showed.

A similar characteristic of higher kurtosis from Kendall’s test was also found when we varied the number of principal components used for the genotype adjustment. As shown in Fig 6(c) and 6(d), kurtosis from Kendall’s test was mesokurtic and much higher than that from Pearson’s test, meaning that Kendall’s test was more robust than Pearson’s test under different genotype encodings. We also found that estimated skewness and kurtosis were insensitive to the number of principal components. Although the difference in skewness between two tests was statistically significant, both skewness were within the range of the moderate symmetry. All these simulation results indicated that Kendall’s test outperformed Pearson’s test in the robustness under different genotype encodings.

### Assessment of suggested method using real data

In addition to the simulated data, we assessed the validity of the suggested method by using real data. The real data were preprocessed by eliminating SNPs and samples (or accessions) that contained missing genotype and/or phenotype. The preprocessed data consisted of 7,551 SNPs across 193 diverse accessions of rice (*O. sativa*) that were phenotyped for 30 traits [21]. A detailed description of real data was given in Section *Acquisition of real data and availability*, in which we described how the preprocessed data can be downloaded. As a results of the association analysis, we provided typical Manhattan plots obtained by two test methods in Supporting information S2 Fig. We also compared the power of Kendall’s test to that of Pearson’s test and again found that Kendall’s test was more powerful than Pearson’s test across different traits, as shown in Supporting information S3 Fig.

**Fig 7:**
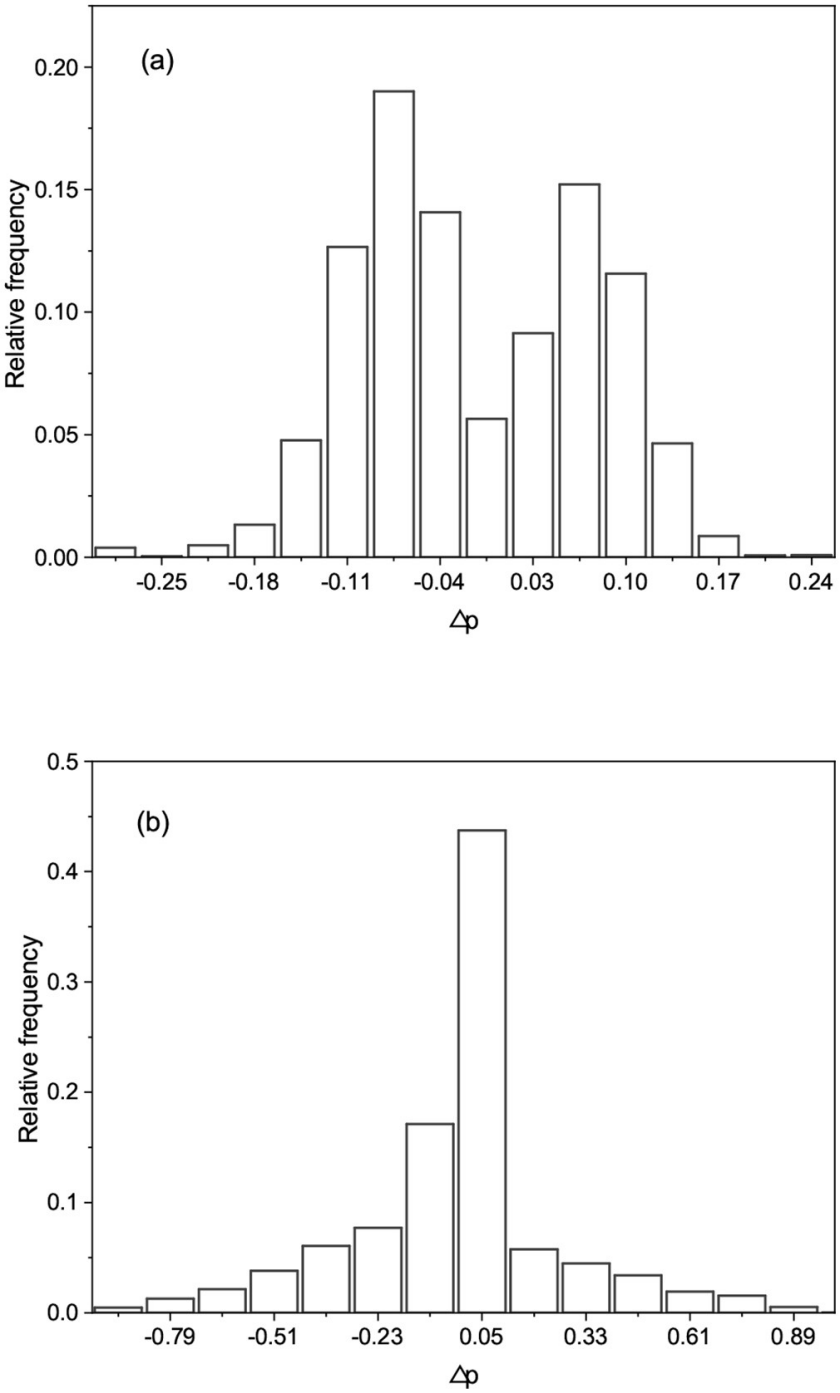
Typical frequency distributions of Δ*p*_*i*_ obtained by (a) Pearson’s test and (b) Kendall’s test for trait *panicle number*. We have used four principal components for the genotype adjustment.

Fig 7 showed a typical frequency distributions of Δ*p*_*i*_ obtained by the two test methods, from which we found similar characteristics to the case of simulated data. Pearson’s test produced the distribution of Δ*p*_*i*_ having two peaks away from zero mean, although the appearance of two peaks was less sharp than the case of simulated data as shown, for example, in Fig 1(a). In contrast, the distribution obtained by Kendall’s test had sharper unimodal compared to the case of simulated data as shown, for instance, in Fig 1(b). This implied that Kendall’s test would be less sensitive to different genotype encodings than Pearson’s test. The frequency distributions of Δ*p*_*i*_ for other traits, each of which was selected from six categories, were given in Supporting information S4 Fig.

**Fig 8:**
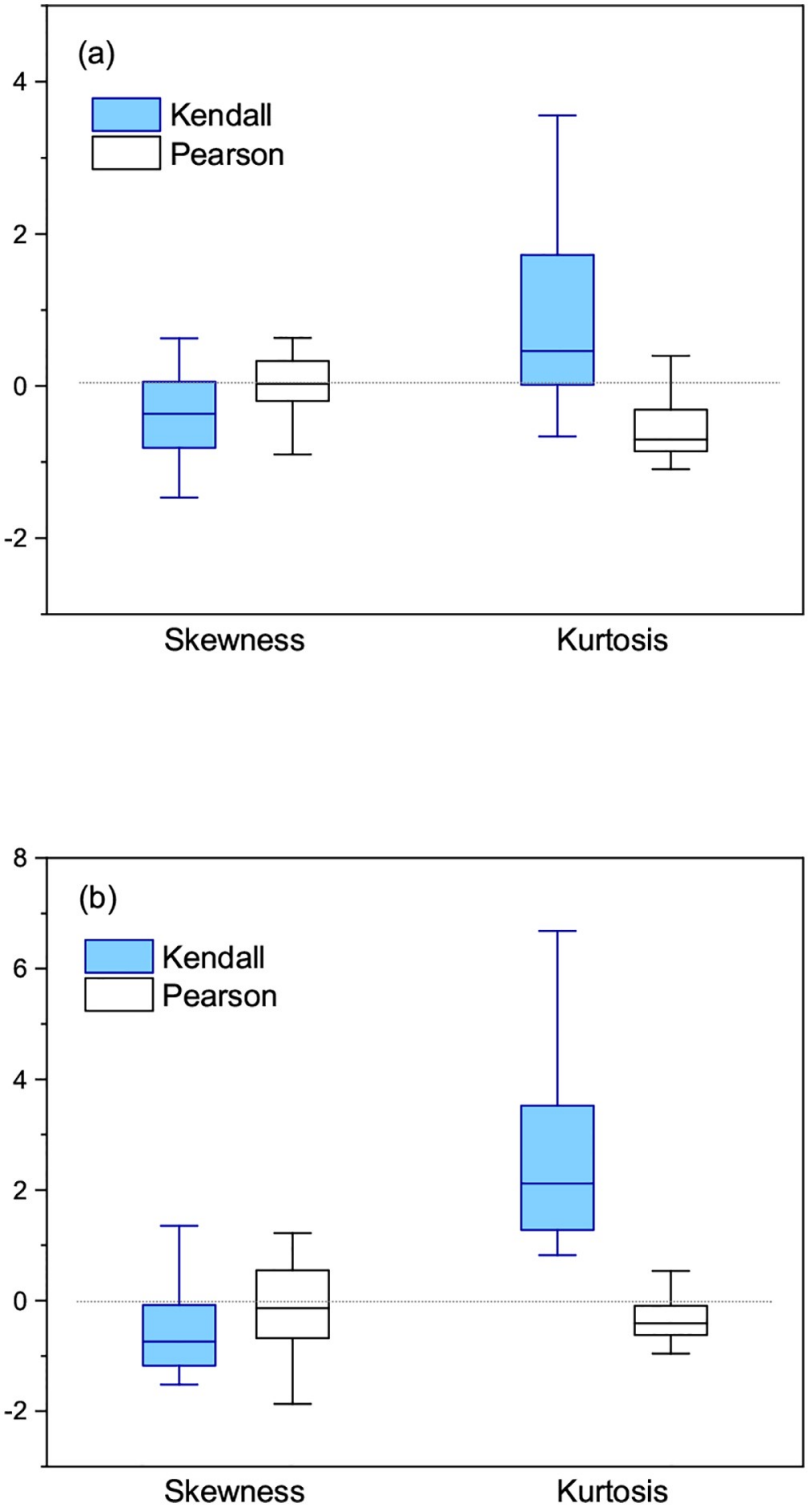
(a) Box plots of skewness and kurtosis obtained by Kendall’s and Pearson’s tests with genotype encodings *E*_1_ = {0, 1, 2} and *E*_2_ = {−1, 0, 1}. (b) Box plots obtained by another pair of genotype encodings *E*_1_ = {1, 2, 3} and *E*_2_ = {2, 3, 4}.

We again quantified the distribution of Δ*p*_*i*_ obtained by Kendall’s and Pearson’s tests in terms of skewness and kurtosis. For each trait, we estimated skewness and kurtosis from Δ*p*_*i*_ of all *m* SNPs *i* = 1, 2, …, *m*. That is, we estimated one pair of skewness and kurtosis for each trait. Fig 8(a) showed box plots of skewness and kurtosis obtained from 30 traits with genotype encodings of *E*_1_ = {0, 1, 2} and *E*_2_ = {−1, 0, 1}. Although skewness estimated by Kendall’s test was relatively low compared to that by Pearson’s test, both skewness are within the range of a symmetric (or moderately symmetric) distribution around zero mean. In contrast, kurtosis estimated from the distributions of Δ*p*_*i*_ obtained by the two methods differed significantly. The distribution obtained by Kendall’s test was leptokurtic (i.e., *G*_2_ > 0), meaning that Δ*p*_*i*_ were more concentrated around zero mean than the case of a normal distribution. On the contrary, the distribution obtained by Pearson’s test was moderately platykurtic (i.e., *G*_2_ < 0). This implied that Kendall’s test was more robust to the different genotype encodings than Pearson’s test. To support the insensitivity of Kendall’s test against different engotype encodings, we took another pair of genotype encodings, *E*_1_ = {1, 2, 3} and *E*_2_ = {2, 3, 4}, and estimated skewness and kurtosis. As Fig 8(b) showed, we obtained similar skewness to the case of Fig 8(a). We also found from Fig 8(b) that Kendall’s and Pearson’s tests produced a positive and a negative kurtosis, respectively. This result demonstrated that the property of skewness and kurtosis was more or less unconnected to different pairs of genotype encodings.

## Discussion

In this study, we addressed the genotype encoding problem in the association study under the assumption that a PCA-based method was used to correct for the population structure. The genotype encoding problem in our case stemmed from the adjustment of categorically (or ordinally) encoded genotypes to continuous values. In principle, any genotype encoding is allowed as long as the encoding scheme can distinguish different genotypes. However, due to the adjustment of an encoded genotype, a test statistic may depend on how a genotype is encoded. In this sense, a desirable method of the association test should possess the robustness of test results under different genotype encodings.

We demonstrated that the Pearson’s test statistic was not invariant under the additive genotype transformation. To alleviate the non-invariance of Pearson’s test, at least partly if not entirely, we suggested a non-parametric test method based on Kendall’s tau. Because Kendall’s association test utilized the relative order in magnitudes of the adjusted genotype values, rather than the values themselves, the test results were more likely to be insensitive to different genotype encodings. In addition, as a non-parametric method, Kendall’s association test did not need an assumption about the phenotype distribution.

This study addressed a statistical method that can be applicable to the topics of interest in population genetics and comparative genomics. In particular, Kendall’s association association test can contribute to finding novel genetic variants that may not be identified by the conventional method in the genome-wide association study. When the population structure is modeled as a fixed effect in linear mixed models, PCA among other methods can be used to correct for the population structure [22]. In such cases, a non-parametric Kendall’s association test can be applied to linear mixed models because numerically adjusted genotypes by PCA in mixed linear models are no longer discrete values and become continuous values. Because the genotype encoding problem can occur other fields, such as genomic selection and breeding, this study may inspire researchers in these fields to investigate not only consistent genotype encoding schemes but also robust prediction methods to different encodings. This study may also bring about a cautious approach to the genotype encoding in the numerical analysis of population genetic data.

The association test based on Spearman’s rank correlation coefficient (or Spearman’s rho) [23] is also a non-parametric test and measures statistical dependence between the rankings of two variables. Although both Kenall’s tau and Spearman’s rho are similar in their theoretical background and robust to outliers, Kendall’s tau is known to be more robust and efficient (i.e., smaller variance) than Spearman’s rho [24]. In addition, while Kendall’s tau is interpretable as the percentage of pairs of variables that show a positive correlation, Spearman’s rho does not have a precise interpretation, These makes Kendall’s tau be the preferable estimator. In terms of the computation, both tests require the same time complexity of *O*(*n* log *n*), where *n* is the sample size [25, 26]. Nevertheless, for the completeness, we provided the results of the test based on Spearman’s rho and compared with those of Pearson’s test in Supporting information S5 Fig.

Genotypes can be grouped into dominant, recessive, and additive genetic models [27]. Although the additive genetic model is known to be underpowered to detect recessive effects [28], the statistical association test with the additive genetic model is a common practice in GWAS regardless of phenotypes. This is because the additive model has reasonable statistical power to detect both additive and dominant effects for the case of categorical genotypes [29]. Certainly, different genetic models can be adopted, in which Pearson’s test is not adequate at least theoretically if not practically. This may lead to investigating whether Kendall’s test is still more robust than Pearson’s test under different genetic model. While the current study did not investigate the robustness of Kendall’s test under different genetic models, the measures, such as skewness and kurtosis, adopted in the study might be also used to analyze the robustness under different genetic models.

Pearson’s test assumes the additive genetic model, ruling out the possibility of a dominant and a recessive genetic model. If an association analysis adopts a non-additive genetic model, such as dominant or recessive genetic model, the normality assumption of the phenotype distribution would not be satisfied. As a result, the test power of a parametric method, such as Pearson’s test, is generally lower than that of a non-parametric method. In such a case, the non-parametric method of Kendall’s test can be a more cautious approach by removing assumptions about the population distribution, such as the normality of the phenotype values. In this sense, the relation between the genetic model and the test method should be understood in future works.

## Materials and methods

### Acquisition of real data and availability

We used an open access resources of rice (*O. sativa*) data set as an assessment of the proposed method. The data set consist of 44,100 SNP variants across 413 diverse accessions (or samples) that were phenotyped for 34 traits [21]. We excluded four qualitative traits from the analysis and used 30 numerical traits for the association test. Table 1 shows the list of traits that we used in this study. The original data set can be downloaded at http://ricediversity.org/data/index.cfm under the title “44K SNP set” (http://ricediversity.org/data/sets/44kgwas/). We preprocessed the data set by eliminating SNPs and samples that contained missing genotype and/or phenotype values. The preprocessed data consisted of 7,551 SNPs across 193 diverse accessions. The codes (R-scripts) and the preprocessed data set used in this study are available at http://github.com/infoLab204/gwas.

**Table 1:** The list of 30 traits that were investigated in this study. Here, ‘BR’ stands for brown rice; ‘PP’ stands for primary panicle; ‘FT’ stands for flowering time; ‘AA’ stands for Arkansas Aberdeen; ‘FA’ stands for Faridpur Aberdeen; ‘S.’ stands for straighthead; ‘P.’ stands for panicle; ‘LW’ stands for length width.

Group	Trait	Group	Trait	Group	Trait
Seed morohology	Seed length	Yield components	P. number per plant	Flowering time	FT at Arkansas
	Seed width		Plant height		FT at Faridpur
	Seed volume		Panicle length		FT at Aberdeen
	Seed surface area		PP branch number		FT ratio of AA
	BR seed length		Seed number per panicle		FT ratio of FA
	BR seed width		Florets per panicle	Morphology	Culm habit
	BR surface area		Panicle fertility		Flag leaf length
	BR volume	Quality	Amylose content		Flag leaf width
	Seed LW ratio		Alkali spreading value	Stress tolerance	S. suseptability
	BR LW ratio		Protein content		Blast resistance

### Generation of simulated data

Following Ref [30], we simulated pairs of genotype and phenotype values to compare the performance of Kendall’s test with that of Pearson’s test. Let *g*_*ij*_ be a matrix of genotypes for SNP *i* and sample *j*, where *i* = 1, 2, …, *m* and *j* = 1, 2, …, *n*. We denote *m* as the number of SNPs and *n* as the sample size. We initially generated *n* pairs of genotype and phenotype for a SNP (say, SNP *i* = 1) and regarded them as the original data. We then sampled a proportion of *n* original genotypes to be permuted randomly while keeping the phenotypes intact. The proportion is called as the shuffling rate *κ*. This generated a new set that includes non-associated genotype-phenotype pairs, and the degree of non-association is proportional to the shuffling rate *κ*. In this way, we could generate both associated and non-associated pairs of genotype and phenotype. We only considered the additive genetic model, on which Pearson’s test is based.

For a given minor allele frequency *q* and under the Hardy-Weinberg model, we generated genotypes and corresponding phenotypes as follows.

1. Set SNP index *i* = 1. Generate *n* genotypes, *g*_*ij*_ ∈ {0, 1, 2} or *g*_*ij*_ ∈ {−1, 0, 1} (*j* = 1, 2, …, *n*) depending on the encoding scheme, with the probabilities of (1 − *q*)^2^, 2*q*(1 − *q*), and *q*^2^, respectively.
2. By using the additive genetic model, evaluate *p*_*j*_ (*j* = 1, 2, …, *n*), the phenotype of the *j*th sample, according to the formula given in Ref [30]:
  $$\begin{array}{c} p_{j}=\sqrt{1-\pi }\;\varepsilon_{j}+g_{ij}\sqrt{\frac{\pi }{2q(1-q)}}\;, \end{array}$$ (2)
  where *π* ∈ [0, 1] is the rate of variation attributable to the quantitative trait and *ε*_*j*_ is a random number from the standard normal distribution, *ε*_*j*_ ∼ *N*(0, 1).
3. For SNP index *i* = 2, 3, …, *m*, generate a proportion of non-associated genotype-phenotype pairs as follows. First, make a duplicate of the original set of genotypes. Second, for a given shuffling rate *κ* ∈ [0, 1], permute randomly selected *κn* genotypes from *n* samples.

Note that as the shuffling rate *κ* increases, a pair of genotype and phenotype is less likely to be statistically associated. This is because *p*_*j*_ in Eq (2) are designed to be statistically associated with *g*_*ij*_.

### Genotype adjustment

Let *g*_*ij*_ be an encoded genotype of SNP *i* (*i* = 1, 2, …, *m*) and sample *j* (*j* = 1, 2, …, *n*). Then, a different genotype encoding could be implemented by a combination of two types of transformations: a multiplicative and an additive transformations. Under both transformations, different genotype encodings from *g*_*ij*_ could be expressed as

$$\begin{array}{c} g_{ij}^{\alpha }=\alpha g_{ij}\;\;\text{and}\;\;g_{ij}^{\beta }=g_{ij}+\beta \;, \end{array}$$ (3)

where *α* and *β* were the multiplicative and the additive factors, respectively. In this way, the same genotype could be encoded as either *g*_*ij*_ or $g_{ij}^{\alpha }$ (and $g_{ij}^{\beta }$), or a combination of the two. In addition, we define the *k*th axis of variation to be the *k*th eigenvector ${\vec{v}}_{k}$ of an *n* × *n* covariance (or correlation) matrix constructed by using *g*_*ij*_. Note that the eigenvectors are invariant under both the multiplicative and the additive genotype transformations of Eq (3).

Using *K* < *n* axes of variations, the adjusted genotype becomes [11, 12],

$$\begin{array}{c} g_{ij}^{adj}=g_{ij}-\sum_{\ell =1}^{n}(\sum_{k=1}^{K}v_{\ell k}v_{jk})g_{i\ell }\;, \end{array}$$ (4)

where *n* is the number of samples and *v*_*ℓk*_ is the *ℓ*th component of the *k*th eigenvector of the covariance matrix.

Under the multiplicative transformation of $g_{ij}^{\alpha }=\alpha g_{ij}$, the adjusted genotype becomes, using Eq (4),

$$\begin{array}{c} g_{ij}^{\alpha ,adj}\equiv {\left(\alpha g_{ij}\right)}^{adj}=(\alpha \;g_{ij})-\sum_{\ell =1}^{n}(\sum_{k=1}^{K}v_{\ell k}v_{jk})(\alpha g_{i\ell }). \end{array}$$ (5)

Thus, under the multiplicative transformation, we have

$$\begin{array}{c} g_{ij}^{\alpha ,adj}=\alpha g_{ij}^{adj}. \end{array}$$ (6)

Similarly, under the additive transformation of $g_{ij}^{\beta }=g_{ij}+\beta$, the adjusted genotype value becomes, using Eq (4),

$$\begin{array}{ccc} g_{ij}^{\beta ,adj} & \equiv & {\left(g_{ij}+\beta \right)}^{adj}=(g_{ij}+\beta )-\sum_{\ell =1}^{n}(\sum_{k=1}^{K}v_{\ell k}v_{jk})(g_{i\ell }+\beta )\\ & = & g_{ij}^{adj}+\beta \Delta_{j}^{K}, \end{array}$$ (7)

where

$$\begin{array}{c} \Delta_{j}^{K}\equiv \{1-\sum_{\ell =1}^{n}(\sum_{k=1}^{K}v_{\ell k}v_{jk})\}. \end{array}$$ (8)

Thus, under the additive transformation, we have

$$\begin{array}{c} g_{ij}^{\beta ,adj}=g_{ij}^{adj}+\beta \Delta_{j}^{K}\neq g_{ij}^{adj}+\beta , \end{array}$$ (9)

unless $\Delta_{j}^{K}=1$.

### Pearson’s test statistics

For each SNP *i* = 1, 2, …, *m*, consider a linear regression model of

$$\begin{array}{c} p_{j}=b_{0}+b\;g_{ij}^{adj}+\varepsilon_{j},\;\;\varepsilon_{j}∼N\left(0,\sigma^{2}\right), \end{array}$$ (10)

where *j* = 1, 2, …, *n*. For each SNP *i*, we test the null hypothesis that $g_{ij}^{adj}$ and *p*_*j*_ are uncorrelated. This corresponds to the null hypothesis of *H*_0_: *b* = 0 against the alternative hypothesis of *H*_1_: *b* ≠ 0 in Eq (10).

Under the null hypothesis, the test statistic of Pearson’s test using the adjusted genotype $g_{ij}^{adj}$ is given as

$$\begin{array}{c} \frac{\hat{b}}{{\text{SE}}_{n}}∼t\left(n-2\right)\;, \end{array}$$ (11)

where

$$\begin{array}{c} {\text{SE}}_{n}=\sqrt{\frac{\sum_{j=1}^{n}{\left(p_{j}-{\hat{p}}_{j}\right)}^{2}}{\left(n-2\right)\sum_{j=1}^{n}{\left(g_{ij}^{adj}-{\bar{g}}_{i}^{adj}\right)}^{2}}}. \end{array}$$ (12)

That is, the test statistics $\hat{b}/{\text{SE}}_{n}$ has a *t*-distribution with (*n* − 2) degrees of freedom. Here, $\hat{b}$ and ${\hat{p}}_{j}$ are given as

$$\begin{array}{c} \hat{b}=\frac{cov({\vec{g}}_{i}^{adj},\vec{p})}{var({\vec{g}}_{i}^{adj})}\;\;\text{and}\;\;{\hat{p}}_{j}=\bar{p}+\hat{b}(g_{ij}^{adj}-{\bar{g}}_{i}^{adj}), \end{array}$$ (13)

where ${\vec{g}}_{i}^{adj}$ and $\vec{p}$ are *n* × 1 row vectors; $\bar{p}$ and ${\bar{g}}_{i}^{adj}$ are the sample means of $g_{ij}^{adj}$ and *p*_*j*_ over *j* = 1, 2, …, *n*, respectively. The invariance of Pearson’s test under different genotype encodings amounts to examining the invariance of the test statistics of Eq (11).

#### Multiplicative genotype transformation

Under the multiplicative genotype transformation, Pearson’s test statistic is given as, according to Eq (11),

$$\begin{array}{c} \frac{{\hat{b}}^{\alpha }}{{\text{SE}}_{n}^{\alpha }}\equiv {\hat{b}}^{\alpha }\sqrt{\frac{\left(n-2\right)\sum_{j=1}^{n}{\left(g_{ij}^{\alpha ,adj}-{\bar{g}}_{i}^{\alpha ,adj}\right)}^{2}}{\sum_{j=1}^{n}{\left(p_{j}-{\hat{p}}_{j}^{\alpha }\right)}^{2}}}. \end{array}$$ (14)

Under the multiplicative genotype transformation, we have

$$\begin{array}{c} {\bar{g}}_{i}^{\alpha ,adj}\equiv \frac{1}{n}\sum_{j=1}^{n}g_{ij}^{\alpha ,adj}=\alpha {\bar{g}}_{i}^{adj},\;\;{\hat{b}}^{\alpha }\equiv \frac{cov({\vec{g}}_{i}^{\alpha ,adj},\vec{p})}{var({\vec{g}}_{i}^{\alpha ,adj})}=\frac{\alpha \;cov({\vec{g}}_{i}^{\;adj},\vec{p})}{\alpha^{2}var\left({\vec{g}}_{i}^{\;adj}\right)}=\frac{1}{\alpha }\;\hat{b}. \end{array}$$ (15)

Using Eq (15), we can express ${\hat{p}}_{j}^{\alpha }$ as

$$\begin{array}{c} {\hat{p}}_{j}^{\alpha }\equiv \bar{p}+{\hat{b}}^{\alpha }(g_{ij}^{\alpha ,adj}-{\bar{g}}_{i}^{\alpha ,adj})=\bar{p}+\frac{1}{\alpha }\hat{b}(\alpha g_{ij}^{adj}-\alpha {\bar{g}}_{i}^{adj})={\hat{p}}_{j}, \end{array}$$ (16)

Put Eqs (15) and (16) into Eq (14), we finally have

$$\begin{array}{c} \frac{{\hat{b}}^{\alpha }}{{\text{SE}}_{n}^{\alpha }}=\frac{1}{\alpha }\;\hat{b}\sqrt{\frac{\left(n-2\right)\alpha^{2}\sum_{j=1}^{n}{\left(g_{ij}^{\;adj}-{\bar{g}}_{i}^{adj}\right)}^{2}}{\sum_{j=1}^{n}{\left(p_{j}-{\hat{p}}_{j}\right)}^{2}}}=\frac{\hat{b}}{{\text{SE}}_{n}}. \end{array}$$ (17)

Thus, Pearson’s test statistics is invariant under the multiplicative genotype transformation.

#### Additive genotype transformation

Similar to the case of the multiplicative transformation, Pearson’s test statistics under the additive genotype transformation can be expressed as

$$\begin{array}{c} \frac{{\hat{b}}^{\beta }}{{\text{SE}}_{n}^{\beta }}={\hat{b}}^{\beta }\sqrt{\frac{\left(n-2\right)\sum_{j=1}^{n}{\left(g_{ij}^{\beta ,adj}-{\bar{g}}_{i}^{\beta ,adj}\right)}^{2}}{\sum_{j=1}^{n}{\left(p_{j}-{\hat{p}}_{j}^{\beta }\right)}^{2}}}, \end{array}$$ (18)

Under the additive genotype transformation, we have from Eq (7),

$$\begin{array}{ccc} & & {\bar{g}}_{i}^{\beta ,adj}\equiv \frac{1}{n}\sum_{j=1}^{n}g_{ij}^{\beta ,adj}={\bar{g}}_{i}^{adj}+\beta {\bar{\Delta }}^{K}, \end{array}$$ (19)

$$\begin{array}{ccc} & & {\hat{b}}^{\beta }\equiv \frac{cov({\vec{g}}_{i}^{\beta ,adj},\vec{p})}{var({\vec{g}}_{i}^{\beta ,adj})}=\frac{\;cov({\vec{g}}_{i}^{\;adj}+\beta {\vec{\Delta }}^{K},\vec{p})}{var({\vec{g}}_{i}^{\;adj}+\beta {\vec{\Delta }}^{K})}=\hat{b},\;\;\; \end{array}$$ (20)

where ${\bar{\Delta }}^{K}$ is the average of Δ^*K*^ in Eq (8) over *n* samples, i.e.,

$$\begin{array}{c} {\bar{\Delta }}^{K}\equiv \frac{1}{n}\sum_{j=1}^{n}\Delta_{j}^{K}. \end{array}$$ (21)

In addition, we have used the fact that $\Delta_{j}^{K}$ is independent of $g_{ij}^{adj}$ and *cov*(*X* + *a*, *Y* + *b*) = *cov*(*X*, *Y*) and *var*(*X* + *a*) = *var*(*X*). Using Eqs (19) and (20), ${\hat{p}}_{j}^{\beta }$ becomes

$$\begin{array}{c} {\hat{p}}_{j}^{\beta }\equiv \bar{p}+{\hat{b}}^{\beta }(g_{ij}^{\beta ,adj}-{\bar{g}}_{i}^{\beta ,adj})={\hat{p}}_{j}+\beta \hat{b}\left(\Delta_{j}^{K}-{\bar{\Delta }}^{K}\right)\;. \end{array}$$ (22)

Put Eqs (19)–(22) together into Eq (18), we finally have

$$\begin{array}{ccc} \frac{{\hat{b}}^{\beta }}{{\text{SE}}_{n}^{\beta }} & = & \hat{b}\sqrt{\frac{\left(n-2\right)\sum_{j=1}^{n}{\left(g_{ij}^{adj}+\beta \Delta_{j}^{K}-{\bar{g}}_{i}^{adj}-\beta {\bar{\Delta }}^{K}\right)}^{2}}{\sum_{j=1}^{n}{\left(p_{j}^{\beta }-{\hat{p}}_{j}^{\beta }\right)}^{2}}}\\ & = & \hat{b}\sqrt{\frac{\left(n-2\right)\sum_{j=1}^{n}{\left\{\left(g_{ij}^{adj}-{\bar{g}}_{i}^{adj}\right)+\beta \left(\Delta_{j}^{K}-{\bar{\Delta }}^{K}\right)\right\}}^{2}}{\sum_{j=1}^{n}{\left\{\left(p_{j}-{\hat{p}}_{j}\right)-\beta \hat{b}\left(\Delta_{j}^{K}-{\bar{\Delta }}^{K}\right)\right\}}^{2}}}\neq \frac{\hat{b}}{{\text{SE}}_{n}}\;, \end{array}$$ (23)

unless *β* = 0. This implies that Pearson’s test statistic is not invariant under the additive genotype transformation.

### Kendall’s test statistics

The Kendall’s statistics tests the null hypothesis *H*_0_: *τ* = 0 against the alternative hypothesis *H*_1_: *τ* ≠ 0. Here, *τ* is the Kendall rank correlation coefficient *τ* (or Kendall’s tau for short), which measures the ordinal association between two sets of quantities. Kendall’s tau quantifies the degree of correlation between two sets of ranked variables (in our case, adjusted genotypes and phenotypes). For given random vectors (*X*, *Y*) and its independent copy $\left(\tilde{X},\tilde{Y}\right)$, Kendall’s tau is defined as

$$\begin{array}{ccc} \tau & \equiv & P\left[\left(X-\tilde{X}\right)\left(Y-\tilde{Y}\right)>0\right]-P\left[\left(X-\tilde{X}\right)\left(Y-\tilde{Y}\right)<0\right]\\ & = & E[sgn\left(X-\tilde{X}\right)\;sgn\left(Y-\tilde{Y}\right)], \end{array}$$ (24)

where *sgn*(⋅) is a sign function, and *P*[⋅] and *E*[⋅] are a probability and an expectation value, respectively. To estimate *τ*, we considered a set of observations *S*_*i*_ for each SNP *i*, each of which consists of pairs of an adjusted genotype and a phenotype denoted as $S_{i}=\left\{\left(g_{ij}^{adj},p_{j}\right),\;j=1,2,\ldots ,n\right\}$. Any pair of observations $\left(g_{ij}^{adj},p_{j}\right)$ and $\left(g_{i\ell }^{adj},p_{\ell }\right)$ are said to be concordant if $g_{ij}^{adj}>g_{i\ell }^{adj}$ and *p*_*j*_ > *p*_ℓ_, or if $g_{ij}^{adj}<g_{i\ell }^{adj}$ and *p*_*j*_ < *p*_*ℓ*_; they are said to be discordant if $g_{ij}^{adj}>g_{i\ell }^{adj}$ and *p*_*j*_ < *p*_ℓ_, or if $g_{ij}^{adj}<g_{i\ell }^{adj}$ and *p*_*j*_ > *p*_*ℓ*_; otherwise they are tied. The estimate of Kendall’s tau is given as

$$\begin{array}{c} {\hat{\tau }}_{n}\equiv \frac{2(n_{c}-n_{d})}{n(n-1)}, \end{array}$$ (25)

where *n* is the number of observations, and *n*_*c*_ and *n*_*d*_ are the numbers of concordant and discordant pairs, respectively. ${\hat{\tau }}_{n}$ is an unbiased estimator of *τ* when the observations are independent and identically distributed. ${\hat{\tau }}_{n}$ takes values $-1\leq {\hat{\tau }}_{n}\leq 1$: ${\hat{\tau }}_{n}=\pm 1$ corresponds to perfect (dis)agreements and ${\hat{\tau }}_{n}=0$ when they are independent.

When *n* → ∞ (practically, n≳20), the following statistics is known to converge asymptotically to the standard normal distribution [31]. That is, when ${\hat{\tau }}_{n}$ is an unbiased estimate of *τ*,

$$\begin{array}{c} \frac{{\hat{\tau }}_{n}-\tau }{\sigma_{n}}∼N\left(0,1\right),\;\text{where}\;\sigma_{n}=\sqrt{\frac{2(2n+5)}{9n(n-1)}}\;. \end{array}$$ (26)

The test is carried out under the null hypothesis of the independence of the genotypes and the phenotypes (i.e., *H*_0_: *τ* = 0). The proposed method has additional advantage of not requiring any assumption about the phenotype distribution. Note that the direct computation of *n*_*c*_ − *n*_*d*_ in Eq (25) involves two nested iterations of the time complexity *O*(*n*^2^). An efficient algorithm for computation *n*_*c*_ − *n*_*d*_ was proposed that was built on the merge sort and had the time complexity of *O*(*n* log *n*) [25].

In the following, we demonstrated that the test statistics Eq (26) is invariant under the multiplicative genotype transformation. As shown in Eq (6), the adjusted genotype under the multiplicative genotype transformation is given as $g_{ij}^{\alpha ,adj}=\alpha g_{ij}^{adj}$. If $g_{ij}^{adj}>g_{ik}^{adj}$, then we have $\alpha \;g_{ij}^{adj}>\alpha \;g_{ik}^{adj}$, which, in turn, gives $g_{ij}^{\alpha ,adj}>g_{ik}^{\alpha ,adj}$. This implies that the ordinal property of the adjusted genotype is maintained under the multiplicative transformation. Thus, the Kendall’s test statistics is invariant under the multiplicative genotype transformation.

### Invariance and non-invariance of test statistics

We denoted $g_{ij}^{adj}$ as the adjusted genotype of *g*_*ij*_ by a PCA-based method, whose detailed expression was given in Eq (4). Under the multiplicative and the additive transformations, the adjusted genotypes became

$$\begin{array}{ccc} g_{ij}^{\alpha ,adj} & \equiv & {\left(\alpha g_{ij}\right)}^{adj}=\alpha g_{ij}^{adj}, \end{array}$$ (27)

$$\begin{array}{ccc} g_{ij}^{\beta ,adj} & \equiv & {\left(g_{ij}+\beta \right)}^{adj}=g_{ij}^{adj}+\beta \Delta_{j}^{K}, \end{array}$$ (28)

where $\Delta_{j}^{K}$ was defined in Eq (8). $\Delta_{j}^{K}$ satisfies $0\leq \Delta_{j}^{K}\leq 1$. It can be thought of as a proportion of the remainder after the genotype adjustment by *K* axes of variation. When *K* = *n*, where *n* is the number of samples, we have used all axes of variation to adjust the genotype, and there is no remainder (i.e., $\Delta_{j}^{K}=0$). When *K* = 0, no genotype adjustment was made (i.e., $\Delta_{j}^{K}=1$). A detailed derivation of Eqs (27) and (28) was given in Section *Genotype adjustment*. Eq (27) demonstrated that the multiplicative transformation and the genotype adjustment were commutative. That is, the order of the multiplicative transformation and the genotype adjustment was interchangeable. This suggested that test results should be invariant under the multiplicative genotype transformation. The test statistics of both Pearson’s and Kendall’s tests were invariant under the multiplicative genotype transformation, as we showed in Sections *Pearson’s test statistics* and *Kendall’s test statistics*, respectively.

In contrast to the multiplicative transformation, the additive genotype transformation and the genotype adjustment were not commutative as shown in Eq (28). This suggested that the Pearson’s test statistic should be dependent on the additive genotype transformation. Eq (23) showed that, unless *β* = 0, the Pearson’s test statistic was not invariant under the additive genotype transformation. This meant that the result of Pearson’s test might depend on the genotype encoding scheme. For example, we would have different test results from two genotype encodings, *g*_*ij*_ = {−1, 0, 1} and $g_{ij}^{\beta }=\left\{0,1,2\right\}$, which were related by *β* = 1.

We next considered the non-invariance of Kenall’s test under the additive genotype transformation. The non-commutativity between the genotype adjustment and the additive transformation suggested that the Kendall’s test statistic might also depend on the additive genotype transformation. Because Kendall’s tau of Eq (24) was based on the rankings of the adjusted genotypes, not the adjusted genotypes themselves, an analytic expression of the changes in the rankings under the additive genotype transformation was not easy to obtain. We instead empirically investigated the behavior of the rankings under the additive transformation. If the relative order in magnitude between a pair of adjusted genotypes was maintained under the additive transformation, then the estimate of Kendall’s tau of Eq (25) would be invariant under the additive transformation. To quantify the degree of the invariance under the additive transformation, we considered the difference between a pair of samples in their adjusted genotype values before and after the additive transformation. We then examined how much the relative order between two adjusted genotype values was preserved under the additive transformation. That is, for a pair of samples *j* and *k*, we defined $\delta_{jk}^{adj}\equiv g_{ij}^{adj}-g_{ik}^{adj}$ and $\delta_{jk}^{\beta ,adj}\equiv g_{ij}^{\beta ,adj}-g_{ik}^{\beta ,adj}$ for each SNP *i*, and investigated how probable $\delta_{jk}^{\beta ,adj}>0$ (or $\delta_{jk}^{\beta ,adj}<0$), given $\delta_{jk}^{adj}>0$ (or, $\delta_{jk}^{adj}<0$).

### Skewness and kurtosis

Skewness and kurtosis are measures for the shape of a frequency distribution. The unbiased estimate of skewness *G*_1_ and kurtosis (or excess kurtosis) *G*_2_ are given as [20]

$$\begin{array}{ccc} G_{1} & \equiv & \frac{\sqrt{n(n-1)}}{n-2}\frac{m_{3}}{m_{2}^{3/2}} \end{array}$$ (29)

$$\begin{array}{ccc} G_{2} & \equiv & \frac{\left(n-1)(n+1\right)}{\left(n-2)(n-3\right)}\frac{m_{4}}{m_{2}^{2}}-3\frac{{\left(n-1\right)}^{2}}{\left(n-2)(n-3\right)}, \end{array}$$ (30)

where *n* is the number of samples. The moments *m*_2_, *m*_3_, and *m*_4_ are given as

$$\begin{array}{c} m_{r}\equiv \frac{1}{n}\sum_{i=1}^{n}{\left(x_{i}-\bar{x}\right)}^{r}\;\;\text{for}\;\;r=2,3,4, \end{array}$$ (31)

where $\bar{x}$ is the sample mean. We set $\bar{x}=0$ in this study because we used the robustness criterion of zero mean (i.e., Δ*p*_*i*_ = 0).

Skewness is a unitless number and measures the degree of symmetry of a distribution. As skewness is closer to zero, the distribution becomes more symmetric. If skewness is greater (less) than zero, then the distribution is called right (left) skewed (i.e., the right tail is longer than the left tail). As a rule of thumb, the distribution is said to be approximately symmetric when the absolute value of skewnes is less than or equal to 0.5.

Like skewness, kurtosis has no units. Distributions with zero kurtosis are called mesokurtic and a normal distribution is a typical example of a mesokurtic distribution. Distributions with positive (negative) kurtosis are called leptokurtic (platykurtic). The traditional interpretation of kurtosis has been in terms of the central peak of the distribution. As kurtosis becomes larger, the distribution has a sharper unimodal around the mean. In addition to the traditional interpretation, an increasing kurtosis is associated not only with the concentration of probability mass around the mean but with occasional values away from the mean [32].

### Power of a hypothesis test

One statistical measure to quantify the possibility to capture more true marker-phenotype association can be the test power, which is defined as the probability that the test rejects the null hypothesis when the alternative hypothesis is true. That is, the test power is the probability of accepting the alternative hypothesis when it is true. One can approximate the *t*-distribution by a normal distribution when the number of sample *n* is large. When the alternative hypothesis is not an equality but the negation of the null hypothesis, one refers to test power against a specific alternative hypothesis.

In the case of Pearson’s test, when the alternative hypothesis is true (i.e., *b* = *θ* ≠ 0), the power is, for a given significance level *α* = 0.05 and the two-sided (or two-tailed) test, given as

$$\begin{array}{ccc} B_{p}\left(\theta \right) & = & P\{\frac{\hat{b}}{{\text{SE}}_{n}}>1.96\;|\;b=\theta \}+P\{\frac{\hat{b}}{{\text{SE}}_{n}}<-1.96\;|\;b=\theta \}\\ & = & 1-P\{\frac{\hat{b}-\theta }{{\text{SE}}_{n}}>1.96-\frac{\theta }{{\text{SE}}_{n}}\;|\;b=\theta \}+P\{\frac{\hat{b}-\theta }{SE_{n}}<-1.96-\frac{\theta }{{\text{SE}}_{n}}\;|\;b=\theta \}\\ & \approx & 1-\Phi (1.96-\frac{\theta }{{\text{SE}}_{n}})+\Phi (-1.96-\frac{\theta }{{\text{SE}}_{n}}), \end{array}$$ (32)

where $\left(\hat{b}-\theta \right)/{\text{SE}}_{n}$ is Pearson’s test statistics in Eq (11) and Φ is the cumulative distribution function of the standard normal distribution. Here, we assumed that for large *n*, $\left(\hat{b}-\theta \right)/{\text{SE}}_{n}$ approximately follows the standard normal distribution. Similarly, for the case of Kendall’s test, the power is given as

$$\begin{array}{c} B_{k}\left(\theta \right)\approx 1-\Phi (1.96-\frac{\theta }{\sigma_{n}})+\Phi (-1.96-\frac{\theta }{\sigma_{n}}), \end{array}$$ (33)

where *σ*_*n*_ is given in Eq (26). Note that *σ*_*n*_ depends only on the number of samples, while *SE*_*n*_ depends both the number of samples and pairs of genotype and phenotype values.

## Supporting information

S1 Fig The frequency distributions of *δ*^*β*, *adj*^ given *δ*^*adj*^ < 0. The frequency distributions of *δ*^*β*, *adj*^ given *δ*^*adj*^ < 0 with the sample size 300 and the number of principal component *K* = 5. The arrow below the abscissa indicates *δ*^*β*, *adj*^ = 0.
(PDF)

S2 Fig Manhattan plots with real data. (a-b) Manhattan plots of trait seed length obtained from (a) Pearson’s test and (b) Kendall’s test. (c-d) Manhattan plots of trait protein content obtained from (c) Pearson’s test and (d) Kendall’s test.
(PDF)

S3 Fig Test power with real data. Plots of estimated power obtained from Kendall’s and Pearson’s tests for trait seed length versus *θ*, specified values of the alternative hypothesis. Note that estimated powers from Pearson’s test were averaged over 20 SNPs and error bars represented the standard deviations, some of which were too small to be seen. Note also that because the power from Kendall’s test depends only on the number of samples for a given *θ*, the estimated power was same for all SNPs.
(PDF)

S4 Fig The frequency distribution of Δ*p*_*i*_ with real data. The frequency distributions of Δ*p*_*i*_ obtained by Pearson’s and Kendall’s tests for six traits, each of which was selected from six categories. The names of selected traits from each category were: (a) *flowering time at Arkansas*, (b) *culm habit*, (c) *panicle length*, (d) *seed volume*, (e) *blast resistance*, and (f) *protein content*.
(PDF)

S5 Fig The results of Spearman’s test. (a-b): Box plots of (a) skewness and (b) kurtosis obtained by Spearman’s and Pearson’s tests versus different minor allele frequencies using the simulated data. (c-d): Box plots of skewness and kurtosis obtained by Spearman’s and Pearson’s tests using the real data with genotype encodings (c) *E*_1_ = {0, 1, 2} and *E*_2_ = {−1, 0, 1}, and (d) *E*_1_ = {1, 2, 3} and *E*_2_ = {2, 3, 4}.
(PDF)
