# Supplementary material for: A consistent approach to the genotype encoding problem in a genome-wide association study of continuous phenotypes
Source: PLoS One. 2020 Jul 15;15(7):e0236139. doi: 10.1371/journal.pone.0236139 (PMC7363099; doi:10.1371/journal.pone.0236139)
Supplement: S1 Fig — The frequency distributions of δβ, adj given δadj < 0 with the sample size 300 and the number of principal component K = 5. The arrow below the abscissa indicates δβ, adj = 0. (PDF) [file pone.0236139.s001.pdf]

S1 Fig: The frequency distribution of $\delta^{\beta,adj}$ given $\delta^{adj} < 0$

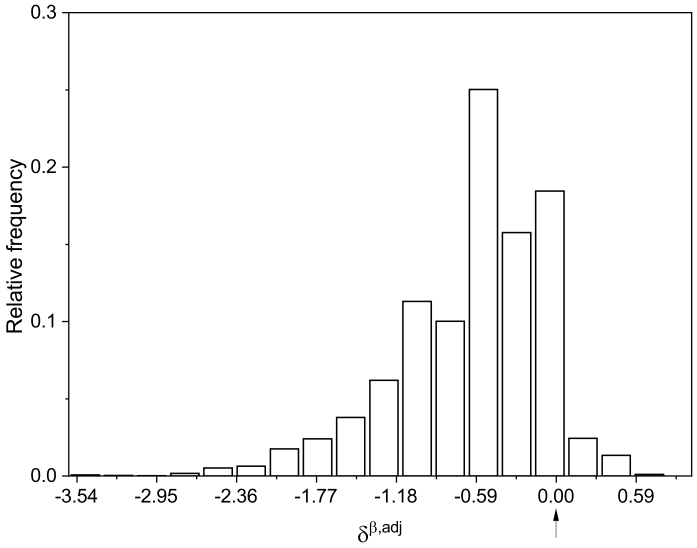

Figure 1: The frequency distributions of $\delta^{\beta,adj}$ given $\delta^{adj} < 0$ with the sample size 300 and the number of principal component $K = 5$. The arrow below the abscissa indicates $\delta^{\beta,adj} = 0$.
