# Supplementary material for: A consistent approach to the genotype encoding problem in a genome-wide association study of continuous phenotypes
Source: PLoS One. 2020 Jul 15;15(7):e0236139. doi: 10.1371/journal.pone.0236139 (PMC7363099; doi:10.1371/journal.pone.0236139)
Supplement: S2 Fig — (a-b) Manhattan plots of trait seed length obtained from (a) Pearson’s test and (b) Kendall’s test. (c-d) Manhattan plots of trait protein content obtained from (c) Pearson’s test and (d) Kendall’s test. (PDF) [file pone.0236139.s002.pdf]

S2 Fig: Manhattan plots with real data

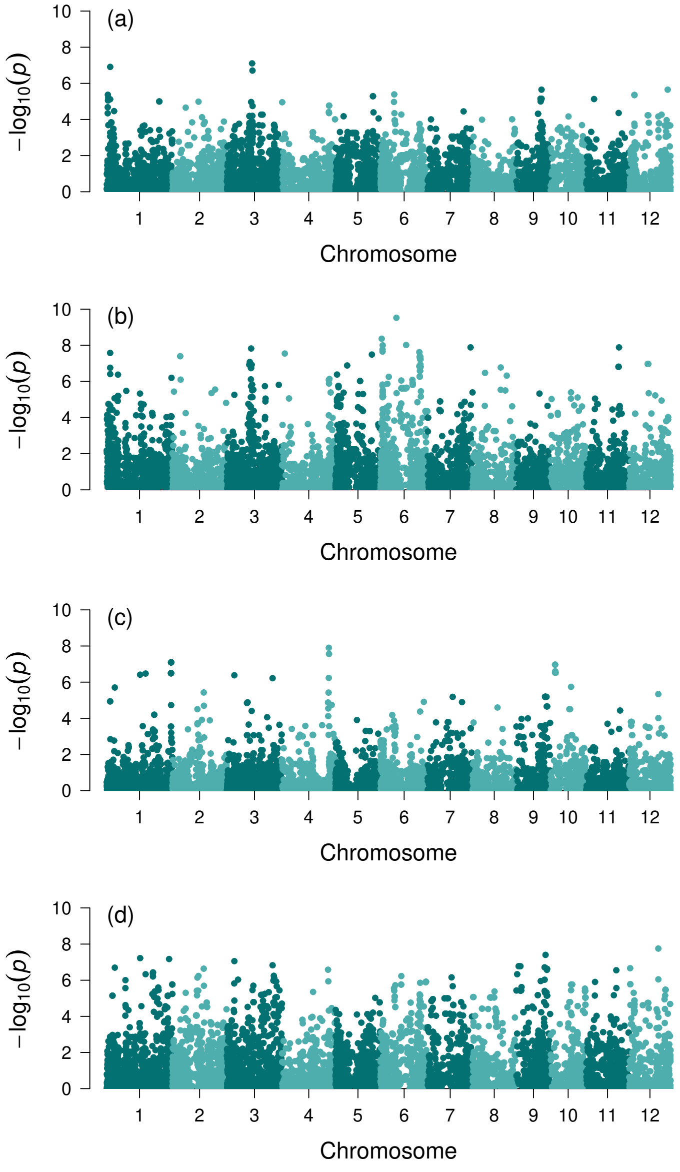

Figure 2: (a-b) Manhattan plots of trait seed length obtained from (a) Pearson's test and (b) Kendall's test. (c-d) Manhattan plots of trait protein content obtained from (c) Pearson's test and (d) Kendall's test.
