# Supplementary material for: A consistent approach to the genotype encoding problem in a genome-wide association study of continuous phenotypes
Source: PLoS One. 2020 Jul 15;15(7):e0236139. doi: 10.1371/journal.pone.0236139 (PMC7363099; doi:10.1371/journal.pone.0236139)
Supplement: S3 Fig — Plots of estimated power obtained from Kendall’s and Pearson’s tests for trait seed length versus θ, specified values of the alternative hypothesis. Note that estimated powers from Pearson’s test were averaged over 20 SNPs and error bars represented the standard deviations, some of which were too small to be seen. Note also that because the power from Kendall’s test depends only on the number of samples for a given θ, the estimated power was same for all SNPs. (PDF) [file pone.0236139.s003.pdf]

S3 Fig: Test power with real data

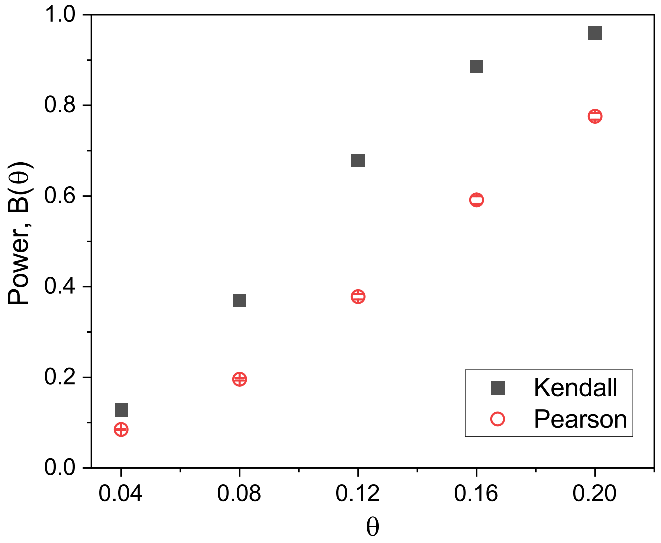

Figure 3: Plots of estimated power obtained from Kendall's and Pearson's tests for trait seed length versus θ, specified values of the alternative hypothesis. Note that estimated powers from Pearson's test were averaged over 20 SNPs and error bars represented the standard deviations, some of which were too small to be seen. Note also that because the power from Kendall's test depends only on the number of samples for a given θ, the estimated power was same for all SNPs.
