# Supplementary material for: A consistent approach to the genotype encoding problem in a genome-wide association study of continuous phenotypes
Source: PLoS One. 2020 Jul 15;15(7):e0236139. doi: 10.1371/journal.pone.0236139 (PMC7363099; doi:10.1371/journal.pone.0236139)
Supplement: S4 Fig — The frequency distributions of Δpi obtained by Pearson’s and Kendall’s tests for six traits, each of which was selected from six categories. The names of selected traits from each category were: (a) flowering time at Arkansas, (b) culm habit, (c) panicle length, (d) seed volume, (e) blast resistance, and (f) protein content. (PDF) [file pone.0236139.s004.pdf]

S4 Fig: The frequency distribution of Δp_i with real data

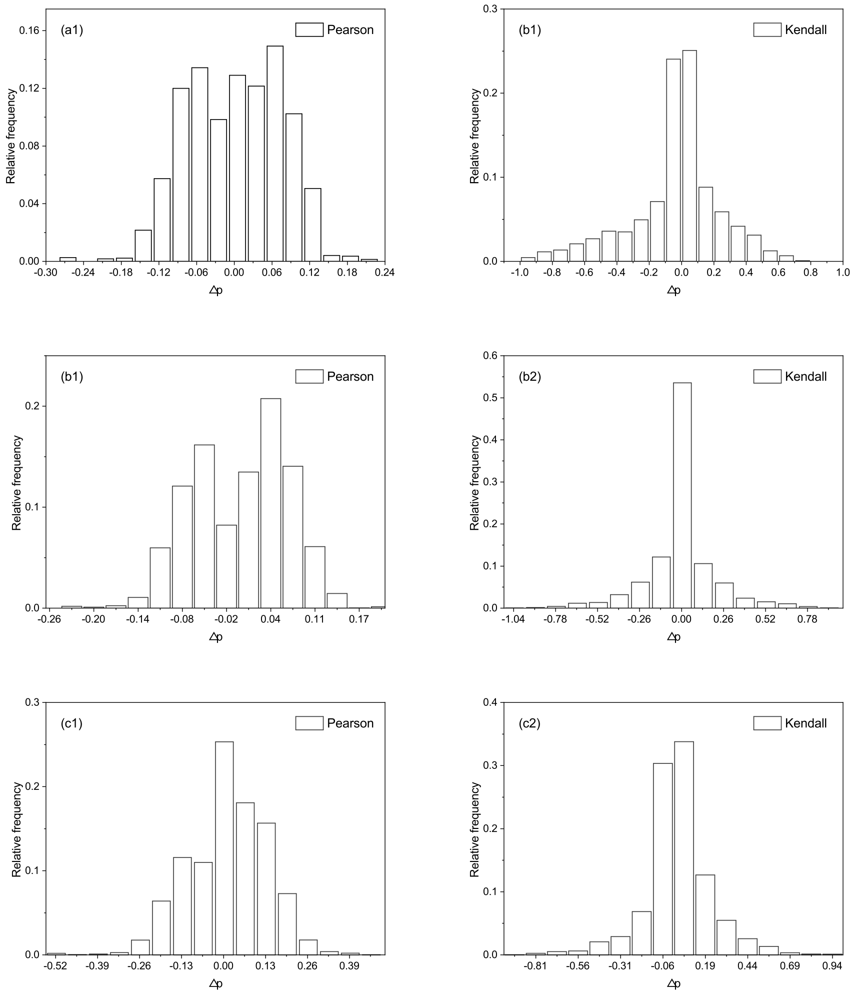

S4 Fig (continued):

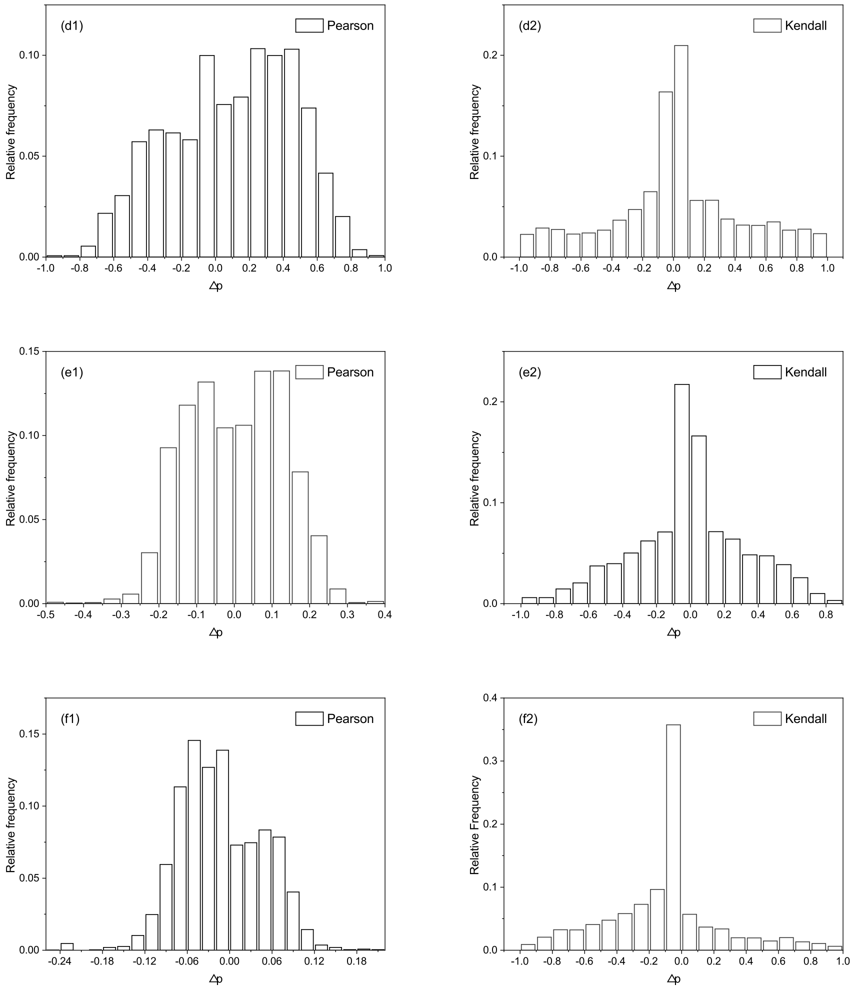

Figure 4: The frequency distributions of Δp_i obtained by Pearson's and Kendall's tests for six traits, each of which was selected from six categories. The names of selected traits from each category were: (a) *flowering time at Arkansas*, (b) *culm habit*, (c) *panicle length*, (d) *seed volume*, (e) *blast resistance*, and (f) *protein content*.
