# Supplementary material for: A consistent approach to the genotype encoding problem in a genome-wide association study of continuous phenotypes
Source: PLoS One. 2020 Jul 15;15(7):e0236139. doi: 10.1371/journal.pone.0236139 (PMC7363099; doi:10.1371/journal.pone.0236139)
Supplement: S5 Fig — (a-b): Box plots of (a) skewness and (b) kurtosis obtained by Spearman’s and Pearson’s tests versus different minor allele frequencies using the simulated data. (c-d): Box plots of skewness and kurtosis obtained by Spearman’s and Pearson’s tests using the real data with genotype encodings (c) E1 = {0, 1, 2} and E2 = {−1, 0, 1}, and (d) E1 = {1, 2, 3} and E2 = {2, 3, 4}. (PDF) [file pone.0236139.s005.pdf]

S5 Fig: The results of Spearman's test

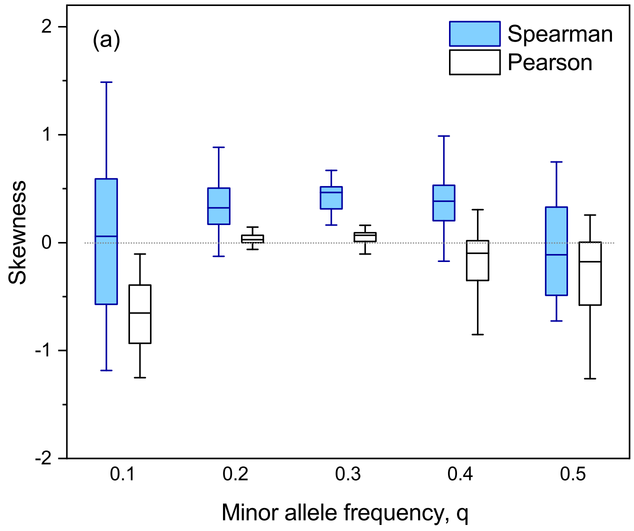

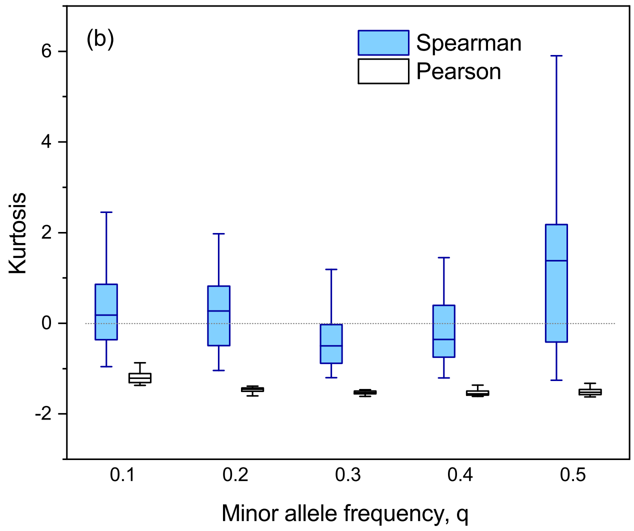

S5 Fig (continued):

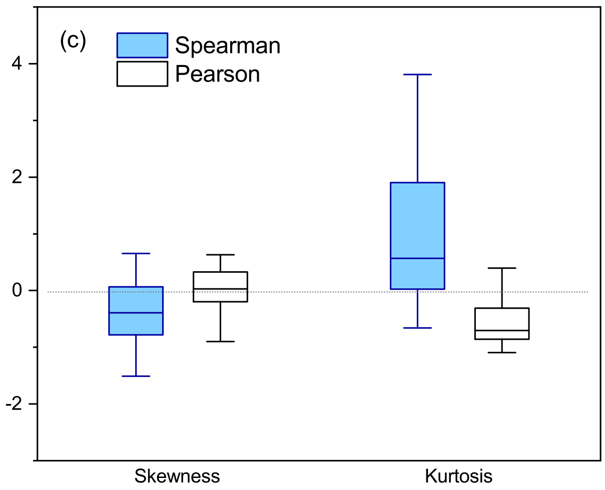

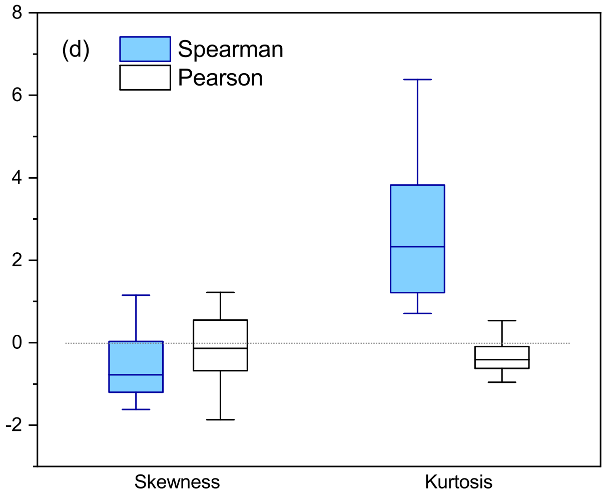

Figure 5: (a-b): Box plots of (a) skewness and (b) kurtosis obtained by Spearman's and Pearson's tests versus different minor allele frequencies using the simulated data. (c-d): Box plots of skewness and kurtosis obtained by Spearman's and Pearson's tests using the real data with genotype encodings (c) $E_1 = \{0, 1, 2\}$ and $E_2 = \{-1, 0, 1\}$, and (d) $E_1 = \{1, 2, 3\}$ and $E_2 = \{2, 3, 4\}$.
